# CAU-63, an Ultramicroporous Al-MOF with a Honeycomb-Shaped 2D IBU

**DOI:** 10.1021/acs.inorgchem.5c03315

**Published:** 2025-10-02

**Authors:** Lasse Wegner, Diletta Morelli Venturi, Evgeniia Ikonnikova, Kai Hetze, Jennifer Theissen, Elien Derveaux, Martin Oschatz, Tom Willhammar, Norbert Stock

**Affiliations:** † Institute for Inorganic Chemistry, Kiel University, Max-Eyth Straße 2, 24116 Kiel, Germany; ‡ Department of Chemistry, 7675 Stockholm University, Svante Arrhenius väg 16 C, 106 91 Stockholm, Sweden; § Helmholtz Institute for Polymers in Energy Applications (HIPOLE Jena), Lessingstrasse 12–14, 07743 Jena, Germany; ∥ Membrane Separations, Adsorption, Catalysis, and Spectroscopy for Sustainable Solutions (cMACS), KU Leuven (Arenberg), Celestijnenlaan 200F, 3001 Leuven, Belgium; ⊥ Institute for Materials Research (Imo-Imomec), Analytical and Circular Chemistry (ACC), NMR Group, 54496 Hasselt University, Agoralaan Building D, 3590 Diepenbeek, Belgium; # Institute for Technical and Environmental Chemistry, Friedrich-Schiller-University Jena, Philosophenweg 7a, 07743 Jena, Germany; ∇ Kiel Nano, Surface and Interface Science KiNSIS, Kiel University, Christian-Albrechts-Platz 4, 24118 Kiel, Germany

## Abstract

The hydrothermal synthesis of the new aluminum metal-organic framework (Al-MOF) **CAU-63** [Al_7_(OH)_12_O_3_(2,4-HPydc)_3_] and two new Al coordination polymers (CPs) **Al-Pydc-CP1** [Al_2_(OH)_5_(2,4-HPydc)] and **Al-Pydc-CP2** [Al­(OH)­(H_2_O)­(2,4-Pydc)] linked by anions of lutidinic acid (pyridine-2,4-dicarboxylic acid, 2,4-H_2_Pydc) is reported. High-throughput investigations of the Al^3+^/2,4-H_2_Pydc/NaOH/H_2_O system were carried out to determine the fields of formation. An increase of the molar ratio of metal to linker was found to be the key parameter for the formation of higher condensed inorganic building units (IBU), changing from dimeric to one- and two-dimensional structures. The crystal structures were determined by 3D electron diffraction with subsequent Rietveld refinement against powder X-ray diffraction data. The pyridine nitrogen atoms of the linker molecules coordinate to aluminum ions in all three compounds, resulting in crystal structures deviating from the typically observed MIL-53 and CAU-10 type frameworks. The coordination polymers **Al-Pydc-CP1** and **Al-Pydc-CP2** contain edge-sharing Al–O/N polyhedra leading to dimeric and helical IBUs, while in **CAU-63**, tetrameric [Al_4_O_14_N_2_] units are bridged by Al^3+^ ions, leading to a honeycomb Al–O–N network with organic moieties interconnecting the layers. This linkage results in channel-like ultramicropores, which are accessible to H_2_O and NH_3_ molecules but too small to adsorb N_2_ and even CO_2_.

## Graphical abstract

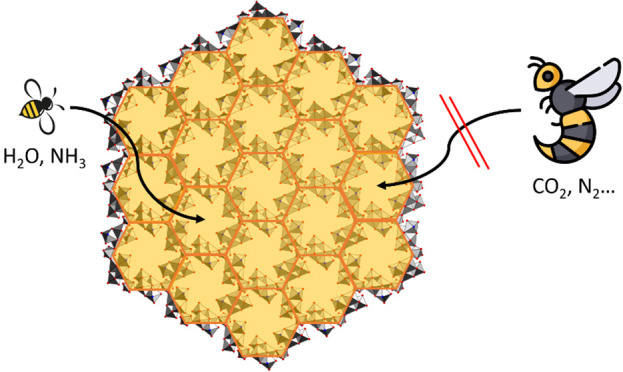

## Introduction

Aluminum is a well-studied element in the field of metal-organic frameworks (MOFs) due to its ubiquity,^1^ low toxicity,^2^ and tendency to form temperature-stable MOFs.^3,4^ Especially in the field of water sorption for atmospheric water harvesting, several highly promising compounds have been reported, e.g., MOF-303,^5^ CAU-10,^6^ or CAU-23.^7^ While the reported Al-MOFs show some structural variety, the vast majority contains chains as inorganic building units (IBUs) constructed from corner-sharing [AlO_6_] polyhedra with different connectivities.^3,8^ In order to expand the range of IBUs and thereby refine the understanding of Al-MOFs, linker molecules with varied coordination modes can be utilized, resulting in novel framework structures and properties.^9–12^ The deprotonated anions of lutidinic acid (pyridine-2,4-dicarboxylic acid) can act as a bidentate chelating ligand, and compounds containing cerium,^13^ manganese,^14^ magnesium,^14^ and cobalt^15^ have been reported. The angle between the coordinating groups in MOF linkers can have a pronounced influence on the structure of the resulting MOF.^8,16^ Therefore, reactions of Al^3+^ salts and lutidinate ions offer the possibility of obtaining coordination compounds with unknown structural motifs. A number of predicted coordination modes that include N–Al–O binding modes are listed in Figure 1.

**Figure 1:**
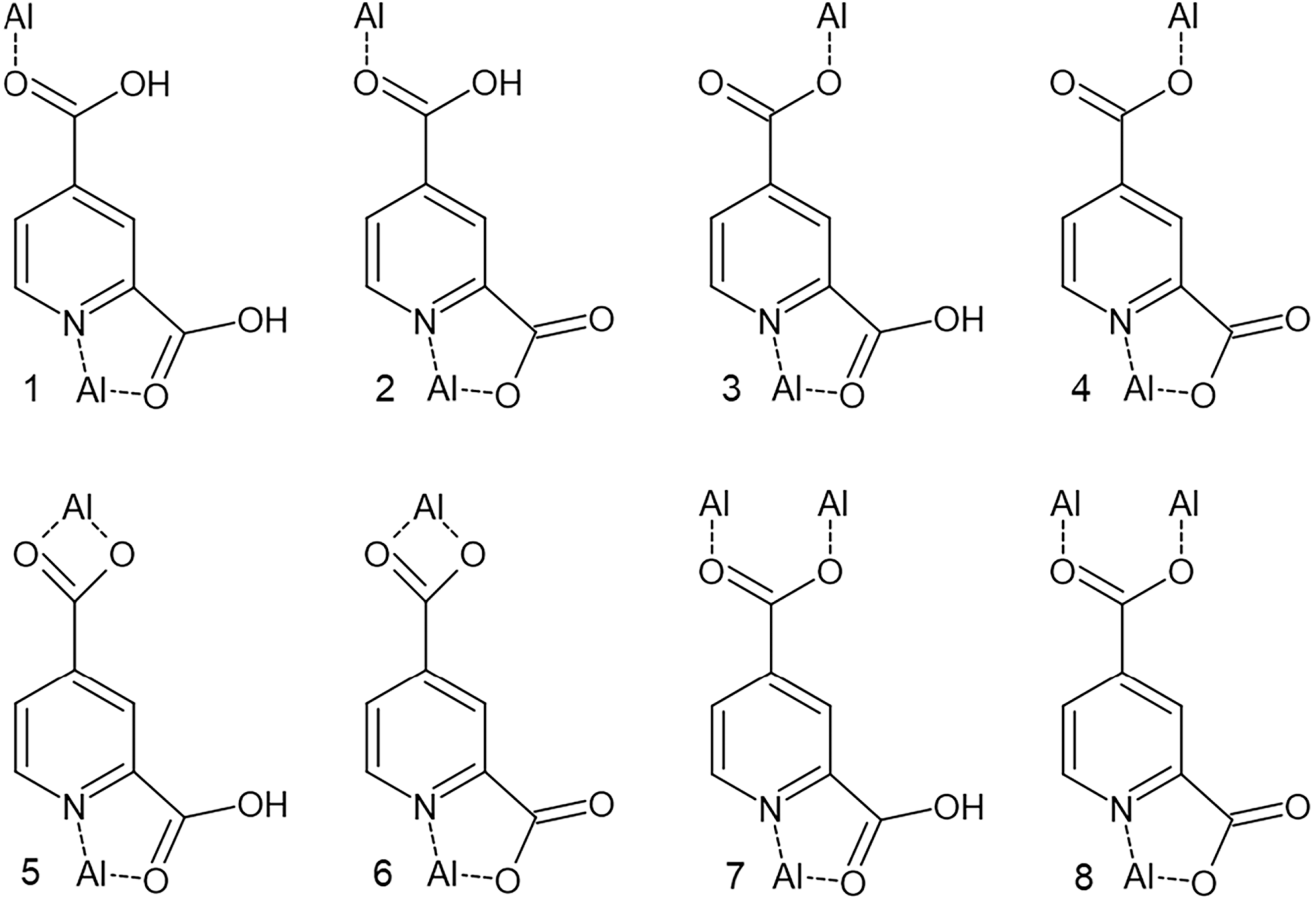
Predicted coordination modes for lutidinic acid and its anions exhibiting the N–Al–O binding mode.

The investigation of novel chemical systems is a laborious and tedious process, necessitating the systematic screening of numerous chemical and process parameters to establish proper synthesis–structure trends. High-throughput (HT) methods based on the concepts of parallelization, miniaturization, and automation have been used to efficiently screen complex reaction systems.^17,18^ HT methods were successfully employed in studies focusing on linker molecules with multiple coordination modes.^19–21^ While discovery and synthesis optimization can thus be accelerated, structural information is often impeded by the limited size of crystallites, which is a prevalent problem in Al-MOF chemistry. Therefore, 3-dimensional electron diffraction (3D ED) has been a valuable method for structure determination of submicron-sized MOF crystals.^22,23^

Here, we report the results of an HT screening of the chemical system Al^3+^/2,4-H_2_Pydc/NaOH/H_2_O that led to the discovery of the new Al-MOF **CAU-63**, [Al_7_(OH)_12_O_3_(2,4-HPydc)_3_], with an unprecedented 2D IBU and two Al coordination polymers (CPs), **Al-Pydc-CP1** [Al_2_(OH)_5_(2,4-HPydc)] and **Al-Pydc-CP2** [Al­(OH)­(H_2_O)­(2,4-Pydc)].

## Experimental Section

All chemicals (AlCl_3_·6H_2_O, Al­(NO_3_)_3_·9H_2_O, Al_2_(SO_4_)_3_·18H_2_O, 2,4-H_2_Pydc, NaOH, and DMF) were obtained from commercial suppliers and used without further purification.

The powder X-ray diffraction (PXRD) data were obtained using a Stoe Stadi P diffractometer in transmission geometry with Cu K_α1_ radiation equipped with a Mythen 1K detector. Water sorption isotherms were collected on a BELSORP-max machine at 293 K. Thermogravimetric (TG) measurements were performed on a Linseis STA 1600 analyzer with a heating rate of 8 K/min in air. Elemental analysis was performed with a vario MICRO cube elemental analyzer from Elementar Analysensysteme GmbH. IR spectra were recorded at room temperature on a Bruker Vertex 70 FT-IR spectrometer using a broadband spectral range extension VERTEX FM for full mid- and far-IR coverage in the range of 6000–80 cm^–1^. Scanning electron microscopy was carried out using a SU8700 scanning electron microscope from Hitachi with an acceleration voltage of 2 kV and EDX analysis was performed with an Ultim Max 100 detector from Oxford Instruments with an acceleration voltage of 10 kV. Optical calorimetry was carried out on a Fraunhofer IWS INFRAsorp.^24^ Each measurement was performed at 299 K and a gas flow rate of 200 mL/min. Ten milligrams of each sample was placed in the sample holder and purged for 600 s with dry nitrogen. The sample mass was maintained constant to eliminate mass influences on heat formation. During adsorption, the samples were exposed to 1000 ppm of NH_3_ in N_2_. Solid-state CP-MAS (cross-polarization magic angle spinning) NMR spectra were acquired at ambient temperature on a JEOL ECZ600R 600 MHz spectrometer (14.1 T) equipped with a 3.2 mm probe. The aromatic signal of hexamethylbenzene was used to calibrate the carbon chemical shift scale (132.1 ppm). The Hartmann–Hahn condition (ω_1H_ = γ_H_B_1H_ = γ_C_B_1C_ = ω_1C_) for cross-polarization was optimized by using the **CAU-63-Cl** sample. Acquisition parameters used for ^13^C NMR were an MAS-rate of 18 kHz, a spectral width of 85 kHz, a 90° pulse length of 2.3 μs, a constant spin-lock field for CP of 70 kHz on the ^1^H channel and ramped CP on the ^13^C channel, a contact time for CP of 2 ms, a recycle delay of 3 and 30 s for **CAU-63-Cl** and lutidinic acid, respectively, an acquisition time of 10 ms and about 3000 accumulations. High-power proton decoupling of 70 kHz during the acquisition time was used for all measurements. 2D ^1^H–^13^C heteronuclear correlation (HETCOR) spectra were recorded using a recycle delay of 30 s, 90° pulse widths of 2.24 and 2.31 μs in the ^1^H and ^13^C channels, respectively, around 10,000 accumulations and identical parameters as described above.

### Synthesis

HT investigations were carried out in a Synthos 3000 microwave reaction system from Anton Paar at a temperature of 135 °C for 6 h with continuous stirring using 4 mL glass reactors. Aqueous solutions of AlCl_3_ (c = 0.5 mol/L) and NaOH (c = 2 mol/L) were employed, and the linker was added first as a solid, followed by the metal salt solution, the base, and finally the water to reach a total volume of 1920 μL. After the reaction, the respective products were obtained as microcrystalline white powders and filtered off, washed three times with water and acetone, respectively, and dried at 80 °C overnight. The molar ratios of 2,4-H_2_PydcAlCl3/NaOH were varied in a broad range with the extreme points of the investigated phase space being 8.75/1.25/1, 1/22/1, and 1/2.625/11.5 (Figure S1). A complete list of all HT-reactions carried out in the discovery and synthesis optimization containing molar ratios of starting materials is given in the Supporting Information (Table S1).

### Optimized Reaction Conditions for CAU-63, Al-Pydc-CP1, and Al-Pydc-CP2

Highly crystalline **CAU-63-Cl** was obtained by mixing 16 mg (96 μmol) of lutidinic acid, 960 μL (480 μmol) of aqueous AlCl_3_ (*c* = 0.5 mol/L), 216 μL (432 μmol) of NaOH (c = 2 mol/L), and 744 μL of H_2_O (yield: 39.5%, based on the amount of lutidinic acid). The same procedure can be followed for the synthesis of **CAU-63-NO**_**3**_ by just exchanging AlCl_3_ with Al­(NO_3_)_3_ (yield: 70.1%, based on the amount of lutidinic acid). **CAU-63-SO**_**4**_ was obtained by mixing 80 mg (479 μmol) of lutidinic acid with 960 μL (480 μmol) of Al_2_(SO_4_)_3_ (*c* = 0.5 mol/L), 400 μL of DMF, and 640 μL of water (yield: 92.8%, based on the amount of Al_2_(SO_4_)_3_). The **CAU-63** compounds contain adsorbed water molecules, which can be removed under a vacuum or at elevated temperatures.

**Al-Pydc-CP1** was synthesized by mixing 16 mg (96 μmol) of lutidinic acid with 288 μL (144 μmol) of aqueous AlCl_3_ (*c* = 0.5 mol/L), 180 μL (360 μmol) of NaOH (*c* = 2 mol/L), and 1452 μL of water (yield: 80.1% based on the amount of aluminum chloride).

**Al-Pydc-CP2** was synthesized by mixing 30 mg (180 μmol) of lutidinic acid with 480 μL (240 μmol) of aqueous AlCl_3_ (*c* = 0.5 mol/L), 210 μL (420 μmol) of NaOH (*c* = 2 mol/L), and 1230 μL of water (yield: 80.8%, based on the amount of lutidinic acid).

### Structure Determination

3D ED data were collected for **CAU**-**63**-**Cl**, **Al-Pydc-CP1**, and **Al-Pydc-CP2** using a JEOL JEM2100 LaB_6_ TEM operated at 200 kV at room temperature, equipped with a Timepix hybrid pixel detector (Amsterdam Scientific Instruments), using the continuous rotation electron diffraction (cRED) method implemented in the software Instamatic.^25^ To prepare a sample for data collection, the powder was crushed in a mortar and dispersed in ethanol; the solution was drop casted onto a lacey carbon-coated copper grid and mounted in a single tilt holder with a high tilt retainer (±80°).

Space group determination was done with the REDp^26^ software, and data integration was performed using the X-ray detector software (XDS).^27^ Structure solution was successfully carried out for **CAU-63-Cl**, **Al-Pydc-CP1**, and **Al-Pydc-CP2** with the 3D ED data using SHELXT-2018 ^28^ implemented in the OLEX2 ^29^ software. It is noted that the water molecules in the pores of **CAU-63-Cl** are removed under a vacuum in the electron microscope. The Rietveld refinements were carried out against PXRD data using Topas Academic V6. For the refinement of **CAU-63-Cl**, a thermally activated sample, containing no water molecules, was used.^30^ Sets of restraints and a rigid body were used to model the crystal structures. The penalties associated with the restraints were initially set high and progressively decreased during the refinement. The models were refined until a convergence was reached.

## Results and Discussion

### Synthesis and Structure Determination

The phase space of the system AlCl_3_/2,4-H_2_Pydc/NaOH/H_2_O was investigated by conducting 211 individual reactions in 4 mL glass reactors in a microwave oven at 135 °C, varying the relative concentrations of the sodium hydroxide, lutidinic acid, and aluminum chloride. This led to the discovery of **CAU-63-Cl** [Al_7_O_3_(OH)_12_(2,4-HPydc)_3_] · 2.4 HCl · 7.7 H_2_O and the two Al-CPs **Al-Pydc-CP1** [Al_2_(OH)_5_(2,4-HPydc)] and **Al-Pydc-CP2** [Al­(OH)­(H_2_O)­(2,4-Pydc)]. All sum formulas were derived from a combination of elemental analysis, thermogravimetric analysis, and IR-spectroscopy and confirmed by the structure models from X-ray diffraction. The chemical formula of **CAU-63-Cl** does not imply the presence of HCl molecules in the pores but rather the presence of H^+^ and Cl^–^ ions; hence, an alternative way of expressing the formula would be H_2.4_[Al_7_O_3_(OH)_12_(2,4-HPydc)_3_]­Cl_2.4_·7.7 H_2_O. Since **CAU-63-Cl** can adsorb water molecules, which can be removed under vacuum or by thermal treatment, the sum formula derived from ED and PXRD data differs by 7.7 H_2_O molecules per formula unit from the one of the samples under ambient conditions. Additionally, the amount of Cl^–^ ions in the pores of **CAU-63-Cl** was quantified by EDX analysis and crystal structure refinement against PXRD data, which led to similar amounts of 2.4 and 2.7 per formula unit, respectively. The section of the ternary diagram containing all three phases is shown in Figure 2, and the whole phase space that was probed can be found in the Supporting Information (Figure S1). The following trends can be extracted from the HT study:

- In contrast to **Al-Pydc-CP1**, fields of formation of **CAU-63-Cl** and **Al-Pydc-CP2** are observed in a large area of the phase space.
- The pH of the reaction mixture is an important parameter. **CAU-63-Cl** forms over a relatively broad pH range from 2.0 to 4.0, **Al-Pydc-CP2** forms predominately at pH values ranging from 1.5 to 2.5, and **Al-Pydc-CP1** is observed exclusively in less acidic conditions with pH values between 4.0 and 5.0.
- A higher molar ratio of metal to linker leads to a higher aluminum content in the products. Thus, **CAU-63-Cl**, **Al-Pydc-CP2**. and **Al-Pydc-CP1** with molar ratios of M/L = 7/3, 2/1, and 1/1 are observed when reaction mixtures with M/L = 5/1, M/L = 3/2, and 4/3 are used. This indicates an increase of the degree of condensation within the structures.

**Figure 2:**
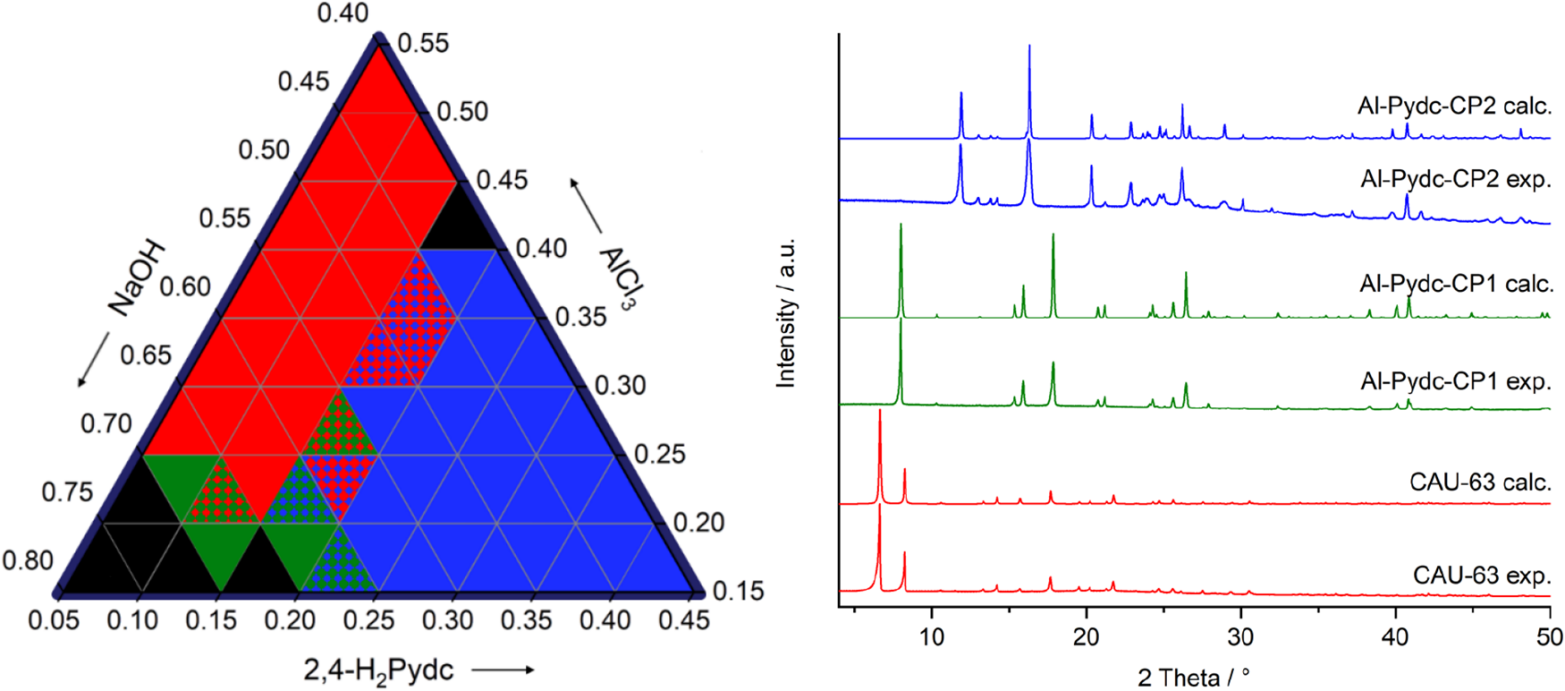
Left: Ternary crystallization diagram showing the influence of molar ratios of NaOH/AlCl_3_/2,4-H_2_Pydc on product formation. All syntheses were conducted at 135 °C for 6 h in 4 mL glass reactors with microwave-assisted heating and water as the solvent. The observed phases are color coded: **CAU-63-Cl** [Al_7_O_3_(OH)_12_(2,4-HPydc)_3_] · 2.4 HCl (red), **Al-Pydc-CP1** [Al_2_(OH)_5_(2,4-HPydc)] (green), and **Al-Pydc-CP2** [Al­(OH)­(H_2_O)­(2,4-Pydc)] (blue), two-colored areas represent phase mixtures. Black areas represent conditions that yielded no crystalline product. Right: Comparison of measured and calculated PXRD patterns **CAU-63-Cl** (red), **Al-Pydc-CP1** (green), and **Al-Pydc-CP2** (blue).

It is possible to synthesize **CAU-63** with Al­(NO_3_)_3_·9H_2_O and Al_2_(SO_4_)_3_·18 H_2_O by replacing AlCl_3_·6 H_2_O, which also leads to microcrystalline products denoted as **CAU-63-NO**_**3**_ and **CAU-63-SO**_**4**_ containing NO_3_^–^ and SO_4_^2–^ ions instead of Cl^–^. The PXRD patterns are very similar, differing mainly in their relative reflection intensities (Figure S3). Since **CAU-63-Cl** shows the highest crystallinity and the highest sorption capacity, the following sections focus on the description of this compound.

### Structure Determination

Since all compounds were obtained as microcrystalline powders, with particle sizes around 1 μm, ab initio structure solution using 3D ED data was carried out for **CAU**-**63**-**Cl**, **Al-Pydc-CP1**, and **Al-Pydc-CP2** and revealed all non-hydrogen atoms of the three phases. The 3D ED structural models were refined against PXRD data through Rietveld refinements. Full details of the refinements and the crystallographic information are given in the Supporting Information (Sections S6 and S7), while the results of the final refinements, including cell parameters and R-factors, are summarized in Table 1.

**Table 1:** Results of the Rietveld Refinements of **CAU-63-Cl**, **Al-Pydc-CP1**, and **Al-Pydc-CP2**

compound	**CAU-63-Cl** [Al_7_(OH)_12_O_3_(2,4-HPydc)_3_]·2.7 HCl	**Al-Pydc-CP1** [Al_2_(OH)_5_(2,4-HPydc)]	**Al-Pydc-CP2** [Al(OH)(H_2_O)(2,4-Pydc)]
empirical formula	C_21_Al_7_N_3_O_27_H_26.7_Cl_2.7_	C_7_Al_2_NO_9_H_9_	C_7_AlNO_6_H_6_
crystal system	trigonal	orthorhombic	monoclinic
space group	*P*3̅	*Pca*2_1_	*C*2/*c*
*a* (Å)	15.2893(4)	13.5097(6)	13.0695(5)
*b* (Å)	15.2893(4)	11.04055(18)	8.7099(3)
*c* (Å)	10.7076(3)	7.31878(14)	15.1655(9)
α (deg)	90	90	90
β (deg)	90	90	101.676(3)
γ (deg)	120	90	90
volume (Å^3^)	2167.70(12)	1091.63(6)	1690.62(14)
*R*_P_ (%)	2.88	3.64	1.63
*R*_WP_ (%)	4.14	5.51	2.39
GoF	0.79	1.14	1.00
*R*_Bragg_ (%)	2.38	1.98	1.53

### Structural Description

The crystal structures of the three crystalline phases differ in the coordination environments of the Al^3+^ ions, dimensionality of the IBU and the resulting coordination polymers as well as the coordination modes of the linker molecules (Figure 3). All Al^3+^ ions are octahedrally surrounded and the first coordination sphere is composed of oxygen atoms from the carboxylate groups, nitrogen atoms of the pyridine ring, O^2–^ and OH^–^ ions as well as H_2_O molecules. Mono and fully deprotonated linker molecules are observed, and in all three compounds, the linker acts as bidentate chelating ligands. The crystallographic assignment of the location of the protons was not possible; therefore, the proposed assignments are based on the spectroscopic results. In all compounds, edge-sharing [AlO_6–*x*_N_*x*_] polyhedra are found and a higher relative aluminum content correlates with a higher degree of condensation of the [AlO_6–*x*_N*_x_*] polyhedra (*x* = 0–2) and a higher dimensional IBU. Some details of the crystal structures are given in the following paragraphs and additional details can be found in the Supporting Information.

**Figure 3:**
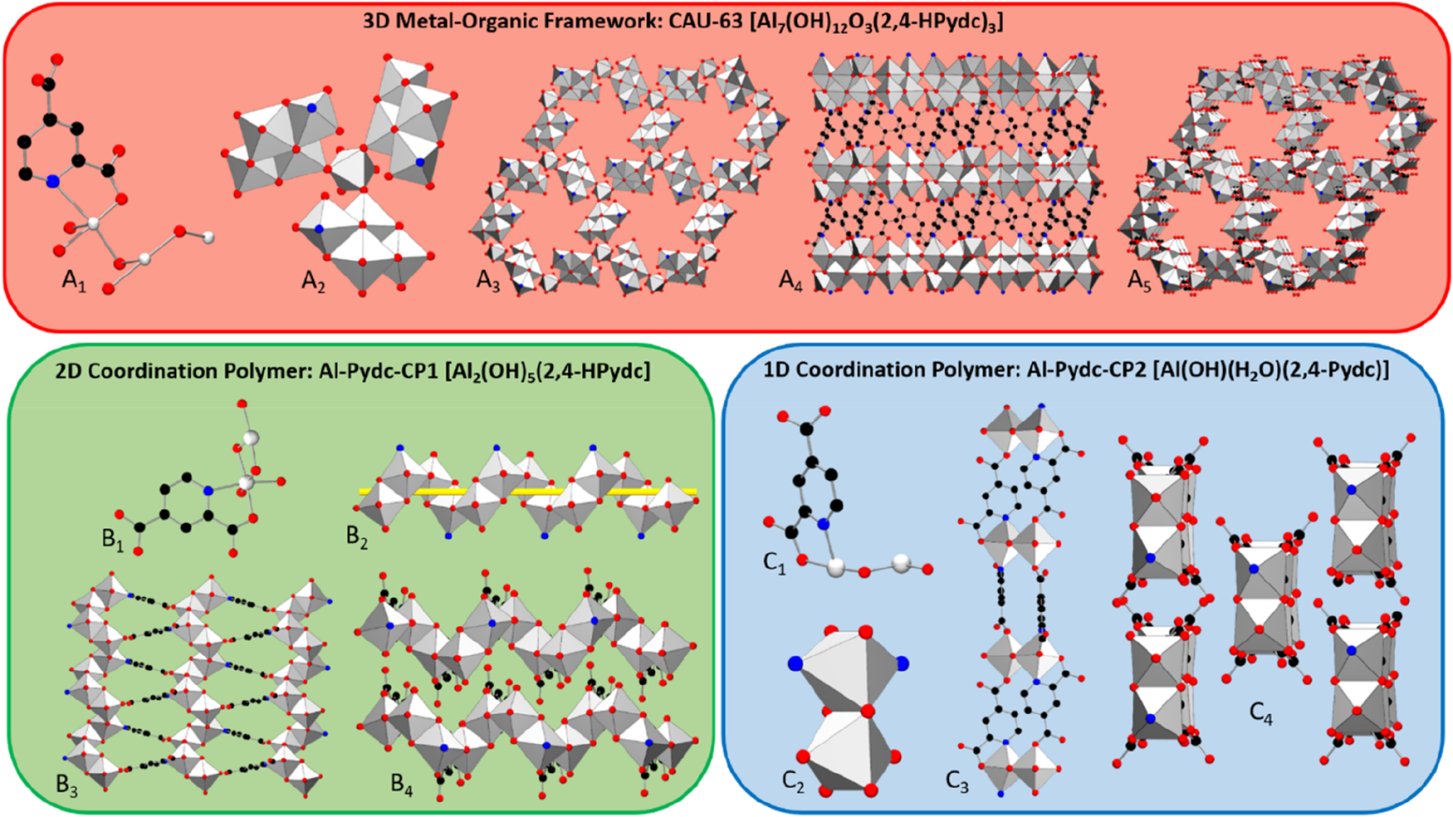
Depiction of the crystal structures with the background colors matching the respective phases in the ternary crystallization diagram (**CAU-63** red, **Al-Pydc-CP1** green, and **Al-Pydc-CP2** blue, Figure 2). **A**_**1**_ **:** Asymmetric unit of **CAU-63**. **A**_**2**_ **:** Tetrameric subunits of the IBU are bridged by Al^3+^ ions. **A**_**3**_ **:** 2D IBU, view along [001]. **A**_**4**_ **:** Interconnection of IBUs by the organic moieties into a 3D framework, view along [010]. **A**_**5**_ **:** View of the 3D framework showing the pores along [001]. **B**_**1**_ **:** Asymmetric unit of the 2D coordination polymer **Al-Pydc-CP1**. **B**_**2**_ **:** Helical IBU with a yellow rod was used for better visualization of the helix. **B**_**3**_ **:** Connection of the IBU via the linker molecules forms a 2D coordination polymer. **B**_**4**_ **:** Stacking of the layers. **C**_**1**_ **:** Asymmetric unit of the 1D coordination polymer **Al-Pydc-CP2**. **C**_2_: Dimeric IBU. **C**_**3**_ **:** Chains formed by connecting the IBUs through the lutidinate ions. **C4:** 3D arrangement of chains. Hydrogen and chlorine atoms have been omitted for the sake of clarity.

The 2D IBU of **CAU-63-Cl**, [Al_7_(OH)_12_O_3_(2,4-HPydc)_3_]·2.4 HCl, is composed of [Al_4_O_14_N_2_] tetramers which are linked into a honeycomb network by an additional corner-sharing of [AlO_6_] octahedron (Figure 3). The layers are stacked along the *c*-axis and interconnected by the lutidinate ions. Each linker is monodeprotonated and coordinates to three Al^3+^ ions through oxygen atoms of the two carboxylate groups and the nitrogen atom of the pyridine ring as seen in coordination mode **7** (Figure 1). Due to the inherent voids in the IBU, a porous structure with one-dimensional channels perpendicular to the IBU is formed. Unduloidal pores along the crystallographic *c*-axis are formed with the IBU as the pore window, limiting the accessibility of the voids. Due to the bottleneck shaped pores it is possible for small molecules to get trapped during the synthesis. Thus, chloride ions are observed occupying the pores, which cannot be removed. For charge balance, protons need to be present. In the dehydrated samples, i.e., those used for structure determination, the protons are likely to bind to the oxygen atoms of the framework. In contrast, in hydrated samples, i.e., those under ambient conditions in air, protons are expected to bind to the water molecules that are present in the pores. To the best of our knowledge, only two other Al-MOFs with 2D IBUs are reported in the literature so far, MIL-96 ^31^ and MIP-213,^32^ making **CAU-63** the third ever Al-MOF to be discovered with this structural feature.

To gain some insights into the degree of protonation of the linker molecules in **CAU-63-Cl**, ^13^C solid-state cross-polarization-magic angle spinning (CP-MAS) NMR spectra of the linker and the MOF were recorded. **CAU-63-Cl** (Figure 4A) exhibits characteristic resonances in the range 120–150 ppm, corresponding to the aromatic carbon atoms within the organic linker and a distinct C=O resonance signal in the carboxylic acid region at 168.9 ppm. Lutidinic acid (Figure 4B), on the other hand, presents an additional carboxylic contribution at 164.6 ppm. This difference is attributed to the dimerization of the lutidinic acid. The position of the 168.9 ppm signal can be explained by linker–linker intermolecular hydrogen bonding via the dimerization of the carboxyl groups. In this case, the C=O carbonyl group is serving as an electron donor, which typically gives rise to a downfield shift (*i*.*e*., to signals at higher ppm values). The 164.6 ppm signal in the linker indicates that not all carbonyl groups are involved in this dimerization process (i.e., they do not act as electron donors) and therefore give rise to a signal at more upfield shifts. This can be confirmed by 2D ^1^H–^13^C HETCOR, (Figure S4), which shows a more intense through-space correlation between the acidic carboxylic protons around 14 ppm and the C=O signal at 168.9 ppm due to hydrogen bonding, compared to the signal at 164.6 ppm.

**Figure 4:**
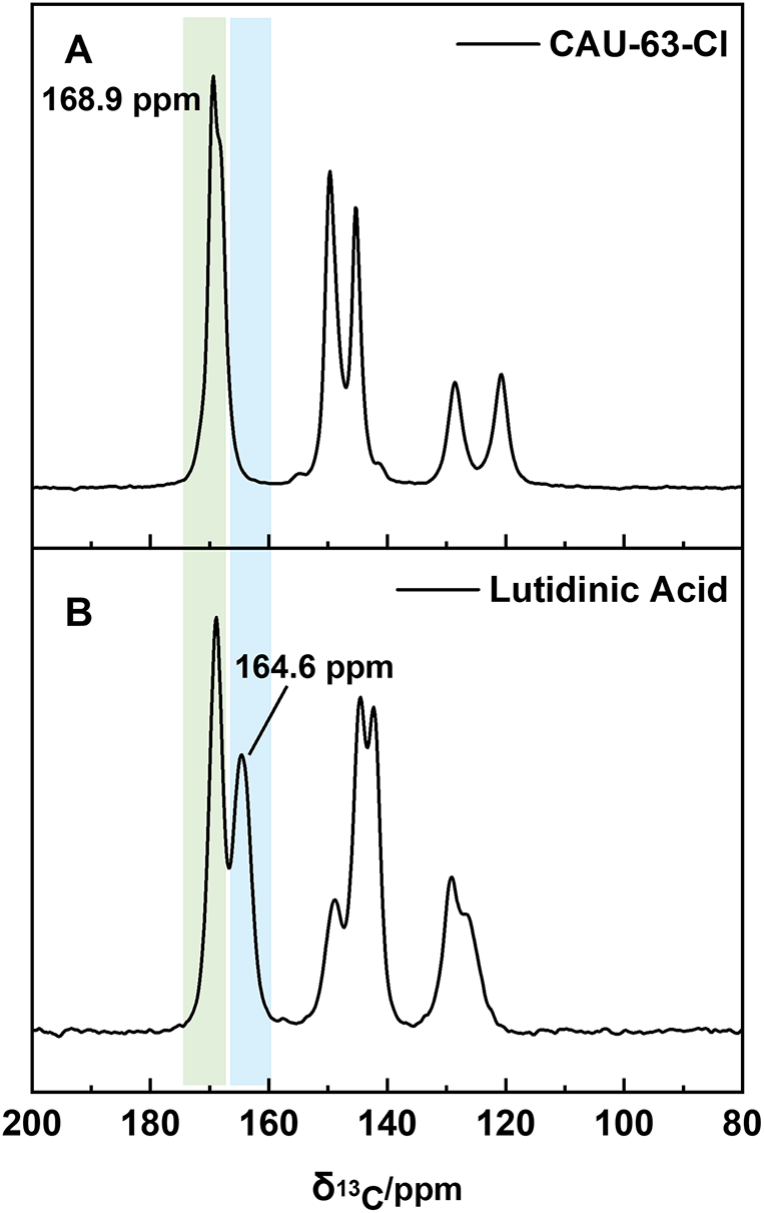
^13^C CP-MAS NMR spectrum of **CAU-63-Cl** (A) and lutidinic acid (B). All carbonyl moieties of **CAU-63-Cl** are engaged in electron-donating interactions, giving rise to only one signal at 168.9 ppm, confirming the presence of monoprotonated linkers and therefore coordination mode 7 (Figure 1).

The signal at 168.9 ppm in **CAU-63-Cl**, therefore, corresponds to a carbonyl group engaged in electron-donating interactions through coordination upon the framework formation of **CAU-63-Cl**, as seen in coordination mode 7 (Figure 1). The absence of the 164.6 ppm signal in **CAU-63-Cl**, which is characteristic of non-electron-donating carbonyl groups in the pristine linker, indicates that all carbonyl moieties in the MOF are participating in coordination. Combining these results with the crystallographic data, this strongly suggests that **CAU-63-Cl** contains a monoprotonated linker with all carbonyl groups engaged in coordination to the aluminum node.

The structure of **Al-Pydc-CP1** contains helical chains of alternating *cis*-edge-sharing [AlO_5_N] and [AlO_6_] octahedra. These chains are connected in the *b-c-*plane through the lutidinate ions, resulting in a 2D coordination network. The handedness of the helices in one layer is the same but alternates in adjacent layers. The linker coordinates with the nitrogen atom of the ring and one of the oxygen atoms of each carboxylate group, leaving two noncoordinating oxygen atoms, as in coordination mode **2** or **3** (Figure 1). The layers are interconnected by hydrogen bonds, as postulated by the O···O distances, to form a dense structure (Figure 3). Only dimers of edge-sharing [AlO_6_] and [AlO_4_N_2_] octahedra that are connected via μ_2_–OH groups are observed as the IBU in **Al-Pydc-CP2** ([Al­(OH)­(H_2_O)­(2,4-Pydc)]). These IBUs are connected via two linker molecules in both directions, forming a 1D coordination polymer. The linker coordinates according to mode **4** (Figure 1) and the coordination sphere of one of the Al^3+^ ions is completed by two terminal H_2_O ligands. The presence of coordinating water molecules is further confirmed by the TG analysis, where a mass loss corresponding to the desorption of water can be seen at around 240 °C (Figure S10). Based on the observed O···O distances, strong hydrogen bonding is anticipated, which leads to the interconnection of the chains to a dense structure (Figure 3). The structure of **Al-Pydc-CP2** is isoreticular to the one observed in [Al_2_(2,5-Pydc)_2_(μ_2_–OH)_2_(H_2_O)_2_], i.e., when the 2,5-pyridinedicarboxylic acid is used as the linker.^33^

The Cl content in the sum formula derived from the Rietveld refinement differs slightly from the one calculated from the EDX measurements with 2.70 Cl versus 2.35 ± 0.26 Cl (Table S2). This can be explained by the disorder of the chloride ions in the material, which makes precise localization and modeling difficult. It can also be explained by small differences in the Cl content of the individual crystallites as well as by the inherent uncertainties of the EDX analysis.

### Sorption Behavior

While coordination polymers **Al-Pydc-CP1** and **Al-Pydc-CP2** have no solvent accessible porosity, **CAU-63** contains 1D pores. Access to the pores is restricted by the small pore aperture of the IBU. The exact pore limiting diameter could not be calculated, since the PXRD data are not sufficient to refine the hydrogen atoms in the structure, and even in the 3D ED data, not all hydrogen atoms could be confidently placed. The electrostatic potential map from the 3D ED refinement suggests a proton bound to the terminal oxygen atom (Al–O) pointing inside the pore, which results in a small pore window (Figures S27 and S28). Sorption experiments show that the pores are not accessible to nitrogen and CO_2_ with kinetic diameters of 3.6 ^34^ and 3.3 Å,^34^ respectively. Water and ammonia molecules with a kinetic diameter of 2.6 ^34^ and 2.9 Å,^34^ respectively, are readily adsorbed already at low partial pressures, which places the limiting pore diameter of **CAU-63** between 3.3 and 2.9 Å. **CAU-63-Cl** shows a type I water sorption isotherm with a maximal uptake of 225 mg/g at 96% relative humidity and 293 K (Figure 5). For **CAU-63-SO**_**4**_ and **CAU-63-NO**_**3**_ the maximum water uptake is reduced to 143 and 162 mg/g, respectively (Figure 5).

**Figure 5:**
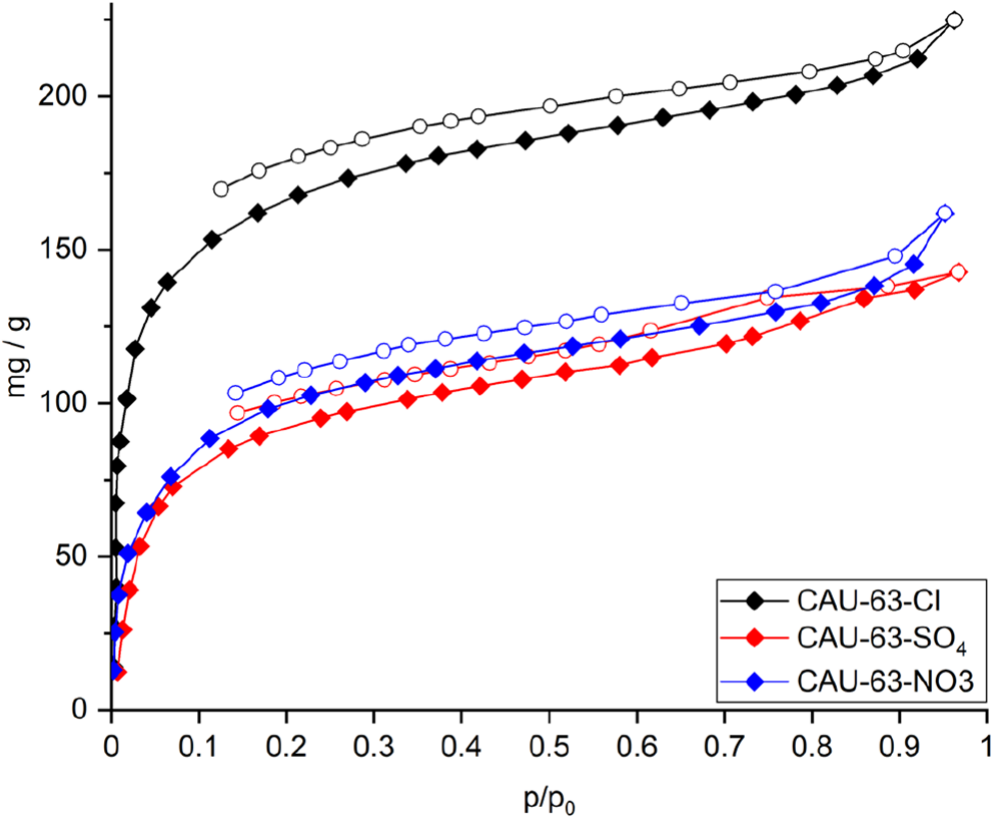
Comparison of the water sorption isotherms of **CAU-63-Cl** (black), **CAU-63-SO**_**4**_ (red), and **CAU-63-NO**_**3**_ (blue) at 293 K.

Additionally, to further probe the accessible pore space of the **CAU-63-X** compounds, the adsorption of NH_3_ with a kinetic diameter of 2.9 Å was investigated with an INFRAsorp optical calorimeter. Ten milligrams of each material was exposed to NH_3_ in nitrogen at a concentration of 1000 ppm, leading to distinct temperature responses (Figure 6).

**Figure 6:**
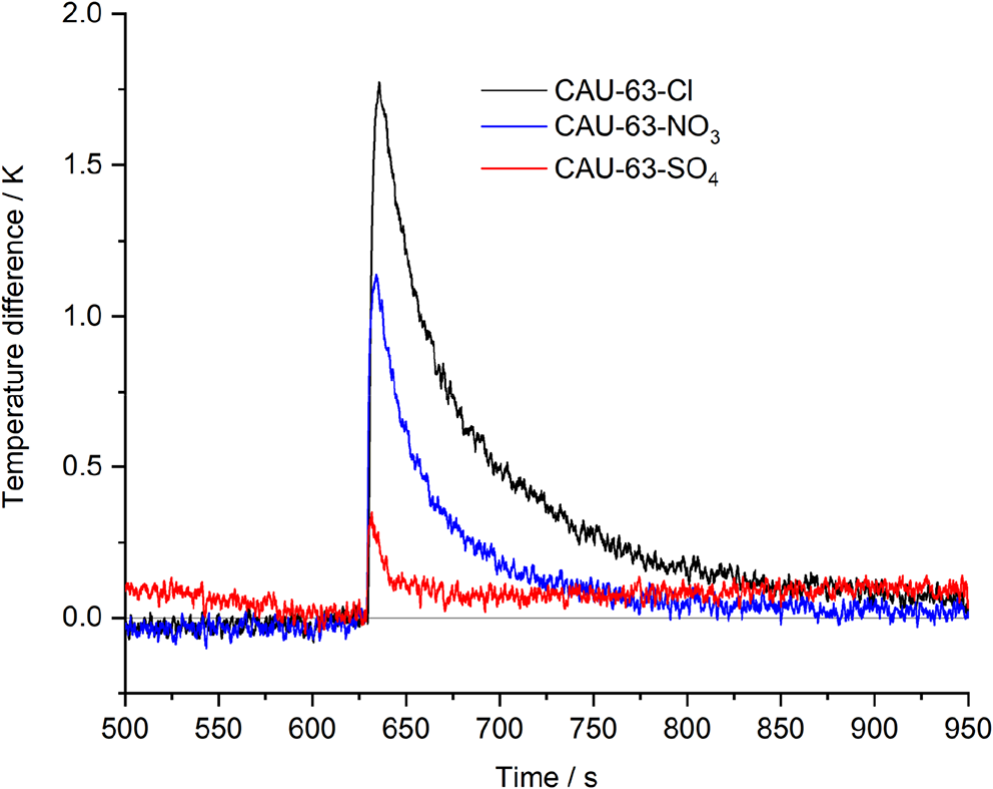
Temperature response of **CAU-63-Cl** (black), **CAU-63-NO**_**3**_ (blue), and **CAU-63-SO**_**4**_ (red) during ammonia adsorption (exposure to 1000 ppm of NH_3_) at 299 K. For better visualization, the raw data were smoothed by adjacent averaging.

All three compounds demonstrate a distinct increase in temperature after contact with ammonia, indicating that the NH_3_ molecules are adsorbed by the materials. Since the heat that is being released during the adsorption process is proportional to the adsorption capacity, these optical calorimetry measurements show the same trend as seen in the volumetric water adsorption, with **CAU-63-Cl** showing the strongest temperature increase, followed by **CAU-63-NO**_**3**_ and finally **CAU-63-SO**_**4**_.

The differing adsorption capacities for water and ammonia between the three modifications are likely caused by the trapping of Cl^–^, HSO_4_^–^/SO_4_^2–^, or NO_3_^–^ ions during the formation of the MOF. The presence of different species in the pores is also reflected in the PXRD data. The relative intensities of especially the first two reflections are greatly influenced by the pore content, as seen in Figure S3.

## Conclusions

A systematic high-throughput investigation using aluminum salts and lutidinic acid led to the discovery of three new coordination polymers. One of the compounds is an MOF denoted as **CAU-63-Cl** with the sum formula [Al_7_(OH)_12_O_3_(2,4-HPydc)_3_] · 2.4 HCl, one a 2D coordination polymer denoted as **Al-Pydc-CP1** with the composition [Al_2_(OH)_5_(2,4-HPydc)], and one a 1D coordination polymer denoted as **Al-Pydc-CP2** with the composition [Al­(OH)­(H_2_O)­(2,4-Pydc)]. Of the new compounds, both **CAU-63** as well as **Al-Pydc-CP1** show IBUs that, to the best of our knowledge, are unprecedented in the aluminum coordination chemistry with **CAU-63** being a rare example of an Al-MOF with 2D IBU. This IBU is a honeycomb-shaped layer with 1D pores, while in **Al-Pydc-CP1**, a helical IBU comprised of edge-sharing [AlO_6_] and [AlO_5_N] polyhedra is found.

The structures of all three compounds were determined by electron diffraction with subsequent Rietveld refinements against powder X-ray diffraction data, and each composition was confirmed by TG and elemental analysis. CP-MAS NMR spectroscopy was employed to elucidate the degree of protonation of the lutidinate ion in **CAU-63-Cl**. Furthermore, the gas uptake properties of **CAU-63** were investigated, and while the pores are too small to adsorb N_2_ and even CO_2_, permanent porosity toward water could be confirmed with a maximum uptake of 225 mg/g at 293 K for **CAU-63-Cl**. Optical calorimetry measurements indicate porosity toward ammonia which places the limiting pore diameter of **CAU-63** between 3.3 and 2.9 Å, which is in good agreement with the structural model obtained from 3D ED.

## Supplementary Material



## Notes

Results from the Rietveld refinements against PXRD data (PDF)
