# Supplementary material for: CAU-63, an Ultramicroporous Al-MOF with a Honeycomb-Shaped 2D IBU
Source: Inorg Chem. 2025 Oct 2;64(40):20254–61. doi: 10.1021/acs.inorgchem.5c03315 (PMC12522134; doi:10.1021/acs.inorgchem.5c03315)
Supplement: Supplementary file 1 [file ic5c03315_si_001.pdf]

Supporting Information

CAU-63, an Ultramicroporous Al-MOF with a Honeycomb Shaped 2D IBU

Lasse Wegner,[a] *Diletta Morelli Venturi,*[ag] *Evgeniia Ikonnikova,*[b] *Kai Hetze,*[c] *Jennifer Theissen,*[d,e] *Elien Derveaux,*[e] *Martin Oschatz,*[c,f] *Tom Willhammar,*[b*] *and Norbert Stock*[ag*]

[a] Institute for Inorganic Chemistry, Kiel University, Max-Eyth Straße 2, 24116 Kiel, Germany.

stock@ac.uni-kiel.de

[b] Department of Chemistry, Stockholm University, Svante Arrhenius väg 16 C, 106 91 Stockholm, Sweden.

tom.willhammar@su.se

[c] Helmholtz Institute for Polymers in Energy Applications (HIPOLE Jena), Lessingstrasse 12-14, 07743. Jena, Germany.

[d] Membrane Separations, Adsorption, Catalysis, and Spectroscopy for Sustainable Solutions (cMACS), KU Leuven (Arenberg), Celestijnenlaan 200F , 3001 Leuven, Belgium.

[e] Institute for Materials Research (Imo-Imomec), Analytical and Circular Chemistry (ACC), NMR group, Hasselt University, Agoralaan Building D, 3590 Diepenbeek, Belgium.

[f] Institute for Technical and Environmental Chemistry, Friedrich-Schiller-University Jena, Philosophenweg 7a, 07743 Jena, Germany.

[g] Kiel Nano, Surface and Interface Science KiNSIS, Kiel University, Christian-Albrechts-Platz 4, 24118 Kiel, Germany.

Table of contents

**S1 Syntheses**	S3-S10
**S2 Solid-state NMR**	S11
**S3 Electron microscopy and EDX**	S11-S15
**S4 Determination of the composition**	S15-S18
**S5 IR-Spectroscopy**	S19-S21
**S6 Structure determination and refinement**	S22-S30
**S7 Details about the crystal structures**	S31-S34
**S8 Sorption**	S35-S36
**S9 Sources**	S37

S1 Syntheses

HT-Investigation

For the discovery of new aluminum lutidinates, reactions were conducted in a Synthos 3000 microwave reaction system from Anton Paar at a temperature of 135 °C for 6 h with continuous stirring. The starting materials were combined in 4 mL glass reactors together with a stirring bar and sealed with a Teflon septum and a plastic screw cap. First, the linker was added, followed by the metal solution, the base and finally the water to reach a filling level of 1920 µL. Both the metal salt as well as the base were employed as aqueous solutions with a concentration of 0.5 mol/L for $AlCl_3$ and 2 mol/L for NaOH, respectively. After the reaction, the respective products were obtained as microcrystalline white powders. They were filtered off, washed three times with water and acetone respectively and dried at 80 °C overnight. For the screening, the relative concentrations of sodium hydroxide, lutidinic acid and aluminum chloride in the reaction mixtures were varied over a wide range as shown in Table 1.

Table S1: Overview of the synthesis conditions used in the HT study. **CAU-63** LC denotes reactions that led to **CAU-63** with a low crystallinity.

Name	2,4-H_2Pydc / µmol	$AlCl_3$ / µmol	NaOH / µmol	Product
HD-L015 A1	180	480	0	Unknown phase
HD-L015 A3	180	480	40.8	Clear solution
HD-L015 A4	180	480	60	Unknown phase
HD-L015 A5	180	480	79.2	Clear solution
HD-L015 A6	180	480	100.8	Clear solution
HD-L016 A1	180	480	120	Clear solution
HD-L016 A2	180	480	139.2	Clear solution
HD-L016 A3	180	480	160.8	Clear solution
HD-L016 A4	180	480	180	Clear solution
HD-L016 A5	180	480	199.2	Clear solution
HD-L016 A6	180	480	220.8	Al-Pydc-CP2
HD-L016 B1	180	480	240	Al-Pydc-CP2
HD-L016 B2	180	480	259.2	Al-Pydc-CP2
HD-L016 B3	180	480	280.8	Al-Pydc-CP2
HD-L016 B4	180	480	300	Al-Pydc-CP2
HD-L016 B5	180	480	319.2	Al-Pydc-CP2
HD-L016 B6	180	480	240.8	Al-Pydc-CP2
HD-L017 A1	180	480	384	Al-Pydc-CP2
HD-L017 A2	180	480	480	NaCl
HD-L017 A3	180	480	576	CAU-63
HD-L017 A5	180	480	768	CAU-63
HD-L017 A6	180	480	864	CAU-63 LC
HD-L017 B1	180	480	960	CAU-63 LC
HD-L017 B2	180	480	1056	CAU-63 LC
HD-L017 B3	180	480	1152	CAU-63 LC
HD-L017 B4	180	480	1248	CAU-63 LC

HD-L017 B5	180	480	1344	Amorphous
HD-L017 B6	180	480	1440	Unknown phase
HD-L018 A1	180	480	504	Clear solution
HD-L018 A2	180	480	528	Amorphous
HD-L018 A3	180	480	552	NaCl
HD-L018 A4	180	480	576	NaCl
HD-L018 A5	180	480	600	CAU-63
HD-L018 A6	180	480	625	CAU-63
HD-L018 B1	180	480	648	CAU-63 LC
HD-L018 B2	180	480	672	CAU-63 LC
HD-L018 B3	180	480	696	CAU-63 LC
HD-L018 B4	180	480	720	CAU-63 LC
HD-L018 B5	180	480	744	CAU-63 LC
HD-L018 B6	180	480	1920	AlO(OH)
HD-L019 A1	208.8	480	624	CAU-63+Al-Pydc-CP2
HD-L019 A2	240	480	624	Al-Pydc-CP2
HD-L019 A3	268.8	480	624	Al-Pydc-CP2
HD-L019 A4	208.8	480	600	CAU-63+Al-Pydc-CP2
HD-L019 A5	240	480	600	Al-Pydc-CP2
HD-L019 A6	268.8	480	600	Al-Pydc-CP2
HD-L020 A1	240	480	640.8	Al-Pydc-CP2
HD-L020 A2	240	480	660	CAU-63 + Al-Pydc-CP2
HD-L020 A3	240	480	679.2	CAU-63 + Al-Pydc-CP2
HD-L020 A4	240	480	700.8	CAU-63 + Al-Pydc-CP2
HD-L020 A5	240	480	720	CAU-63 + Al-Pydc-CP2
HD-L020 A6	240	480	739.2	CAU-63 + Al-Pydc-CP2
HD-L020 B1	240	480	760.8	CAU-63 + Al-Pydc-CP2
HD-L020 B2	240	480	780	CAU-63 + Al-Pydc-CP2
HD-L020 B3	240	480	799.2	CAU-63 + Al-Pydc-CP2
HD-L020 B4	240	480	820.8	CAU-63 + Al-Pydc-CP2
HD-L020 B5	240	480	840	CAU-63 + Al-Pydc-CP2

HD-L020 B6	240	480	859.2	CAU-63 + Al-Pydc-CP2
HD-L021 A1	240	480	900	CAU-63 LC
HD-L021 A2	240	480	940.8	CAU-63 LC
HD-L021 A3	240	480	979.2	CAU-63 LC
HD-L021 A4	240	480	1020	CAU-63 LC
HD-L021 A5	240	480	1060.8	CAU-63 LC
HD-L021 A6	240	480	1099.2	CAU-63 LC
HD-L021 B1	240	480	1140	CAU-63 LC
HD-L021 B2	240	480	1180.8	CAU-63 LC
HD-L021 B3	240	480	1219.2	CAU-63 LC
HD-L021 B4	240	480	1260	CAU-63 LC
HD-L021 B5	240	480	1300.8	CAU-63 LC
HD-L021 B6	240	480	1339.2	CAU-63 + Al-Pydc-CP1
HD-L022 A1	168	480	576	CAU-63
HD-L022 A2	168	480	600	CAU-63
HD-L022 A3	168	480	624	CAU-63
HD-L022 A4	144	480	576	CAU-63
HD-L022 A5	144	480	600	CAU-63
HD-L022 A6	144	480	624	CAU-63
HD-L022 B1	120	480	576	CAU-63
HD-L022 B2	120	480	600	CAU-63
HD-L022 B3	120	480	624	CAU-63
HD-L022 B4	96	480	576	CAU-63
HD-L022 B5	96	480	600	CAU-63
HD-L022 B6	96	480	624	CAU-63
HD-L023 A1	288	480	1560	Al-Pydc-CP1
HD-L023 A2	120	480	1560	Amorphous
HD-L023 A3	288	480	1680	Al-Pydc-CP1
HD-L023 A4	120	480	1680	AlO(OH)
HD-L023 A5	96	480	840	CAU-63 LC
HD-L023 A6	96	480	432	CAU-63
HD-L023 B1	180	240	420	Al-Pydc-CP2
HD-L023 B2	180	240	216	Al-Pydc-CP2
HD-L023 B3	240	240	360	Al-Pydc-CP2
HD-L023 B4	240	240	120	Clear solution
HD-L023 B5	240	120	360	Al-Pydc-CP2
HD-L023 B6	240	120	120	Al-Pydc-CP2
HD-L025 A1	180	120	120	Al-Pydc-CP2 + Unknown Phase
HD-L025 A2	180	120	240	Al-Pydc-CP2
HD-L025 A3	180	120	360	Al-Pydc-CP2

HD-L025 A4	180	120	480	Al-Pydc-CP1+Al-Pydc-CP2
HD-L025 A5	180	120	600	AlO(OH)
HD-L025 A6	180	120	720	AlO(OH)
HD-L025 B1	180	60	120	Al-Pydc-CP2
HD-L025 B2	180	60	240	Al-Pydc-CP2
HD-L025 B3	180	60	360	Amorphous
HD-L025 B4	180	60	480	AlO(OH)
HD-L025 B5	180	60	600	Clear solution
HD-L025 B6	180	60	720	Clear solution
HD-L026 A1	30	210	0	Amorphous
HD-L026 A2	90	150	0	Amorphous
HD-L026 A3	120	120	0	Amorphous
HD-L026 A4	150	90	0	Unknown phase
HD-L026 A5	180	60	0	Amorphous
HD-L026 A6	210	30	0	Amorphous
HD-L026 B1	30	210	24	Amorphous
HD-L026 B2	90	150	24	Amorphous
HD-L026 B3	120	120	24	Amorphous
HD-L026 B4	150	90	24	Amorphous
HD-L026 B5	180	60	24	Amorphous
HD-L026 B6	210	30	24	Amorphous
HD-L045 B1	360	240	60	Clear solution
HD-L045 B2	360	240	120	Clear solution
HD-L045 B3	360	240	180	Clear solution
HD-L045 B4	360	240	240	Clear solution
HD-L045 B5	360	240	300	Al-Pydc-CP2
HD-L045 B6	360	240	360	Al-Pydc-CP2
HD-L045 C1	360	240	420	Al-Pydc-CP2
HD-L045 C2	360	240	480	Al-Pydc-CP2
HD-L045 C3	360	240	540	Al-Pydc-CP2
HD-L045 C4	360	240	600	Al-Pydc-CP2
HD-L045 C5	360	240	660	Al-Pydc-CP2
HD-L045 C6	360	240	720	Al-Pydc-CP2
HD-L045 D1	360	240	780	Al-Pydc-CP2
HD-L045 D2	360	240	840	Al-Pydc-CP2
HD-L045 D3	360	240	900	Al-Pydc-CP2
HD-L045 D4	360	240	960	Al-Pydc-CP1+Al-Pydc-CP2
HD-L045 D5	360	240	1020	Al-Pydc-CP1
HD-L045 D6	360	240	1080	Al-Pydc-CP1
HD-L046 A1	240	48	60	Unknown phase
HD-L046 A2	240	48	120	Unknown phase

HD-L046 A3	240	48	180	Amorphous
HD-L046 A4	240	48	240	Al-Pydc-CP2
HD-L046 A5	240	48	300	Amorphous
HD-L046 A6	240	48	360	Amorphous
HD-L046 B1	240	24	60	Amorphous
HD-L046 B2	240	24	120	Amorphous
HD-L046 B3	240	24	180	Amorphous
HD-L046 B4	240	24	240	Amorphous
HD-L046 B5	240	24	300	Amorphous
HD-L046 B6	240	24	360	Amorphous
HD-L059 A1	240	252	960	Amorphous
HD-L059 A2	168	252	1032	Amorphous
HD-L059 A3	96	252	1104	Amorphous
HD-L059 A4	276	252	924	Al-Pydc-CP1
HD-L059 A5	252	336	948	Al-Pydc-CP1 + Al-Pydc-CP2
HD-L059 A6	294	336	906	CAU-63+Al-Pydc-CP2
HD-L059 B1	336	336	864	Al-Pydc-CP2
HD-L059 B2	376.8	336	823.2	Al-Pydc-CP2
HD-L059 B3	312	444	888	Al-Pydc-CP2
HD-L059 B4	432	444	768	Al-Pydc-CP2
HD-L059 B5	516	444	684	Al-Pydc-CP2
HD-L059 B6	276	1032	924	CAU-63
HD-L059 C1	156	1032	1044	CAU-63
HD-L059 C2	528	252	672	Al-Pydc-CP2
HD-L059 C3	564	252	636	Al-Pydc-CP2
HD-L059 C4	600	252	600	Al-Pydc-CP2
HD-L059 C5	156	1320	1044	CAU-63
HD-L059 C6	420	576	780	Al-Pydc-CP2
HD-L059 D1	468	576	732	Al-Pydc-CP2
HD-L059 D2	372	576	828	Al-Pydc-CP2
HD-L059 D3	276	444	924	CAU-63 + Al-Pydc-CP1
HD-L059 D4	276	912	924	CAU-63
HD-L059 D5	120	552	1080	CAU-63
HD-L059 D6	120	696	1080	CAU-63
HD-L072 A1	240	330	150	Al-Pydc-CP2
HD-L072 A2	210	390	120	Al-Pydc-CP2
HD-L072 A3	150	510	60	Clear Solution
HD-L072 A4	120	570	30	Clear Solution
HD-L072 A5	210	480	30	Clear Solution
HD-L072 A6	120	480	60	Clear Solution

HD-L072 B1	60	510	150	Clear Solution
HD-L072 B2	30	570	120	Clear Solution
HD-L072 B3	30	660	30	Clear Solution
HD-L072 B4	30	480	210	Clear Solution
HD-L072 B5	60	420	240	Clear Solution
HD-L072 B6	120	390	210	Al-Pydc-CP2
HD-L072 C1	300	390	30	Amorphous
HD-L072 C2	150	330	240	Al-Pydc-CP2
HD-L072 C3	210	300	210	Al-Pydc-CP2
HD-L072 C4	240	240	240	Al-Pydc-CP2
HD-L072 C5	390	300	30	Linker
HD-L072 C6	195	105	60	Amorphous
HD-L072 D1	480	210	30	Amorphous
HD-L072 D2	420	150	150	Amorphous
HD-L072 D3	240	60	60	Linker
HD-L072 D4	255	30	75	Amorphous
HD-L072 D5	570	120	30	Linker
HD-L072 D6	330	150	240	Al-Pydc-CP2

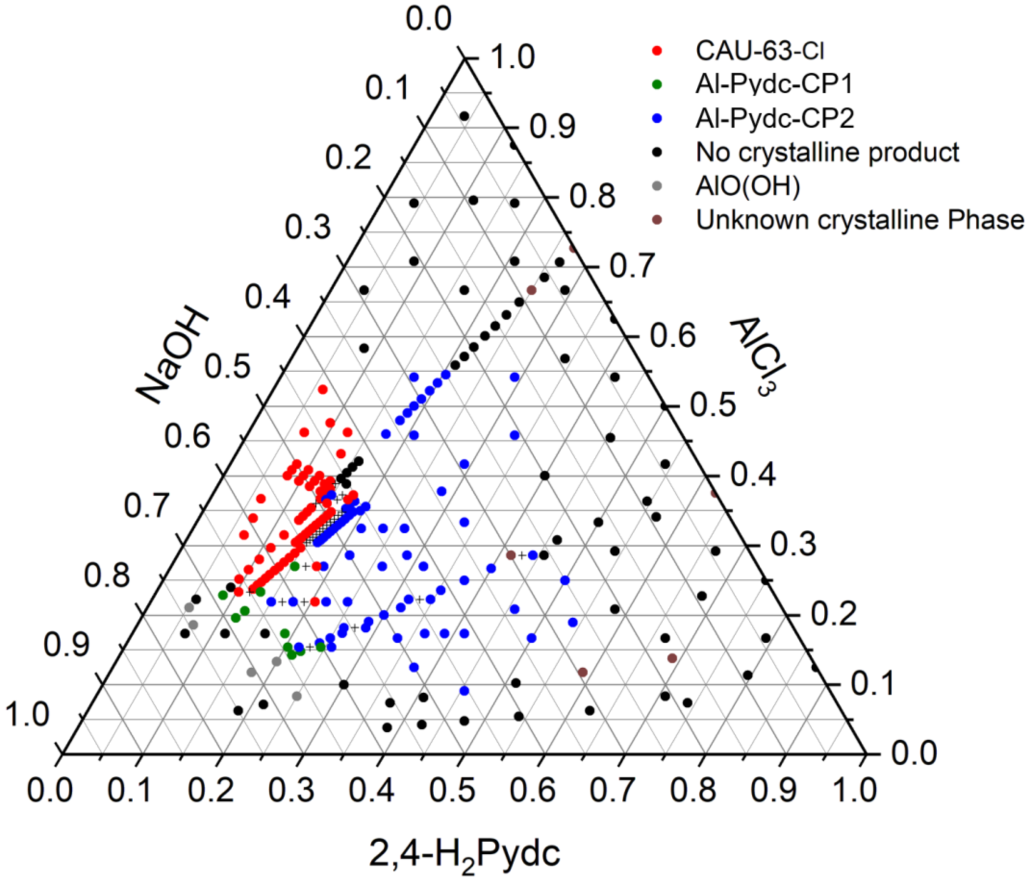

Figure S1: Ternary crystallization diagram of the investigated phase space. The position of the circles shows the relative quantities of sodium hydroxide, aluminum chloride and lutidinic acid that were employed, while the color shows the product that was obtained according to the

legend on the top right. If one reaction led to more than one product, the obtained phases are depicted as multiple circles separated by a plus symbol.

To investigate the impact of the work up procedure on the obtained products, PXRD patterns of the products of the optimized syntheses of **CAU-63-Cl**, **Al-Pydc-CP1** and **Al-Pydc-CP2** before the workup were collected. Additionally, the pre-workup PXRD patterns of the products of three syntheses that were amorphous after the washing steps were measured.

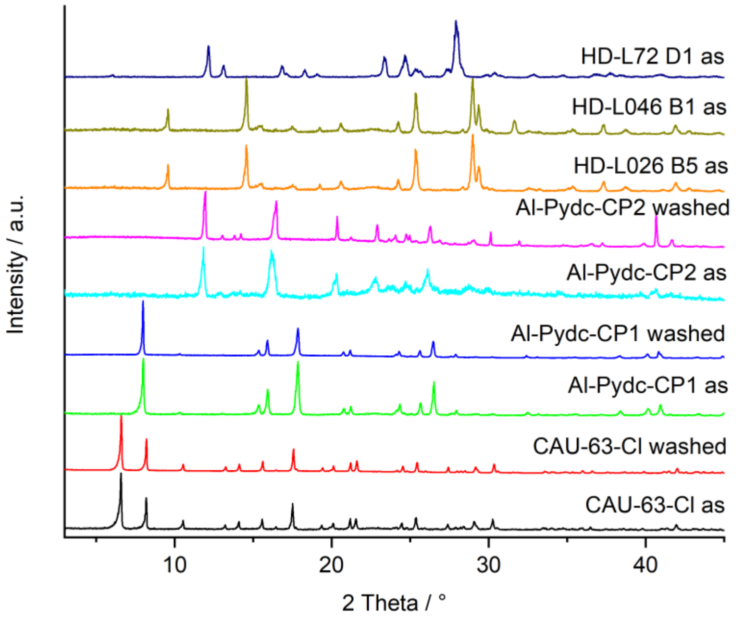

Figure S2: Comparison of the PXRD patterns of **CAU-63-Cl**, **Al-Pydc-CP1** and **Al-Pydc-CP2** before and after the workup procedure as well as the PXRD patterns of the products of selected synthesis products that were amorphous after the work up. As-synthesized products before the work up steps are marked by “as”.

Figure S2 shows, the workup procedure has only a minor effect on the powder patterns of the obtained products for the optimized syntheses, since no additional reflections can be seen in the powder patterns of the as-synthesized products. For the previously amorphous products, crystalline white powders could be obtained before the washing step. Since they were soluble in acetone and exhibited their most intensive reflection in their respective powder patterns at angles higher than 25 ° 2 θ, they were deemed out of the scope of this article and not further investigated.

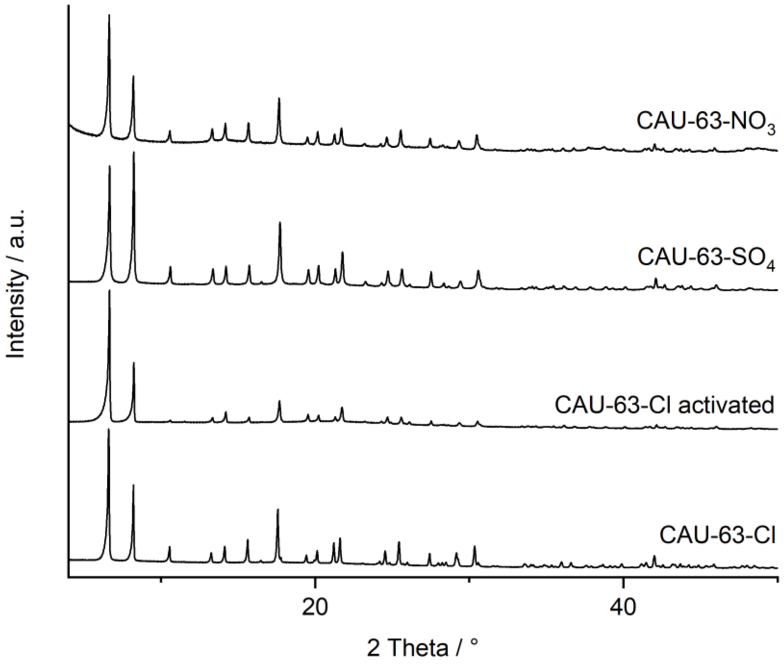

Figure S3: PXRD patterns of **CAU-63** synthesized with different aluminum salts as well as the PXRD pattern of **CAU-63-Cl** that was synthesized with $AlCl_3$ and subsequently activated under vacuum and elevated temperature to remove all adsorbed solvent molecules.

S2 Solid-state NMR spectroscopy

^1H-^{13}C solid-state HETCOR NMR spectrum of lutidinic acid was recorded to determine the degree of protonation of the linker molecules in **CAU-63-Cl**.

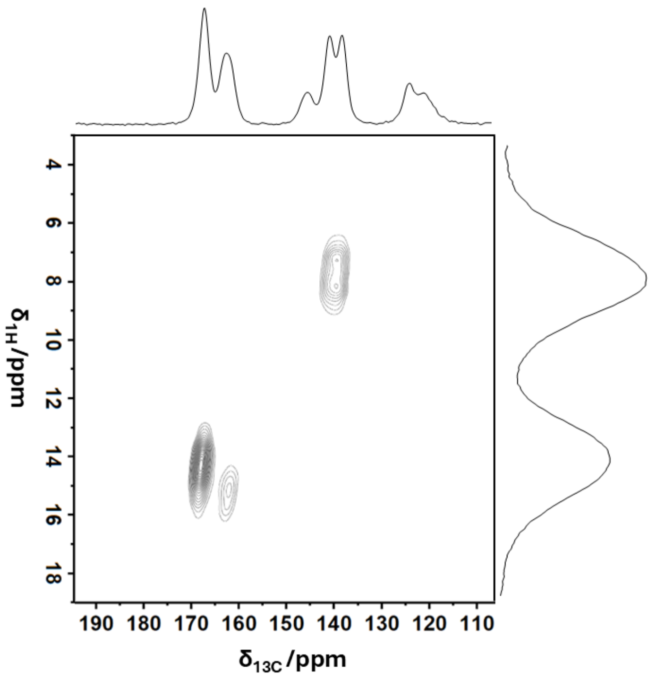

Figure. S4: ^1H-^{13}C solid-state HETCOR NMR spectrum of lutidinic acid. The observation of a strongly increased intensity of the correlation between the carboxylic ^{13}C signal at 168.9 ppm and the ^1H signal at 14 ppm indicates the presence of intermolecular hydrogen bonds. Note that only the correlations of the intense aromatic ^{13}C signals with aromatic ^1H signals are visible due to the variable efficiency of Cross-polarization (*i.e.*, 2D HETCOR is based on cross-polarization of ^1H magnetization to ^{13}C nuclei via spin diffusion).

S3 Electron microscopy and EDX

The SEM micrographs of the three **CAU-63** compounds all show intergrown crystals in the shape of hexagonal prisms. The crystallites in **CAU-63-SO4** are especially agglomerated, while **CAU-63-NO3** shows the most regularly formed crystals.

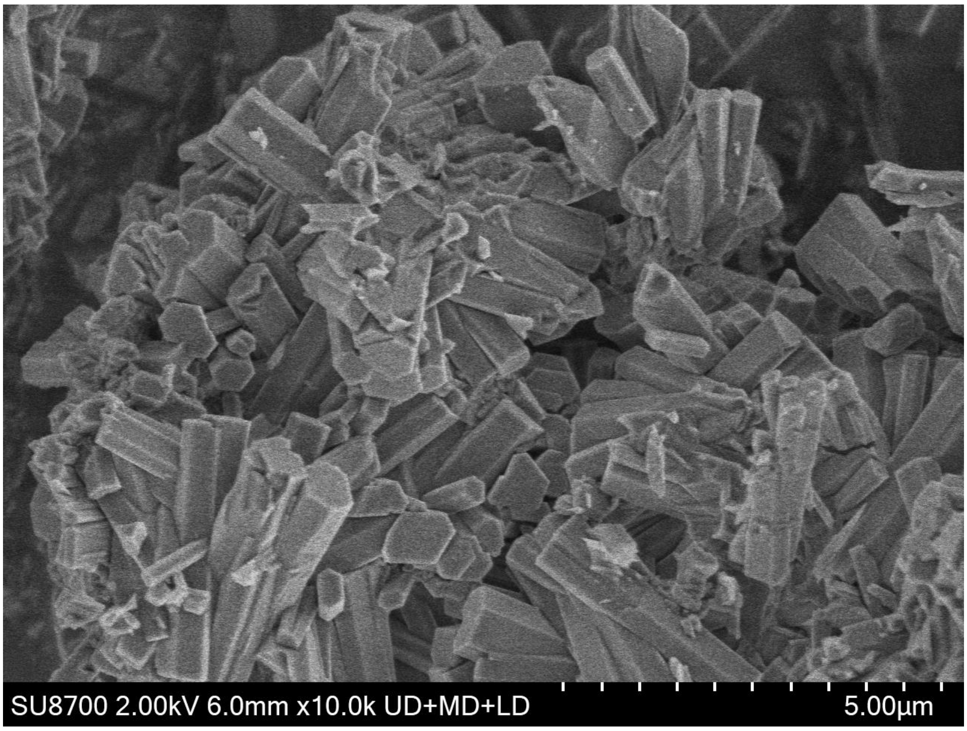

Figure S5: Scanning electron micrograph of **CAU-63-Cl** at a magnification factor of 10K.

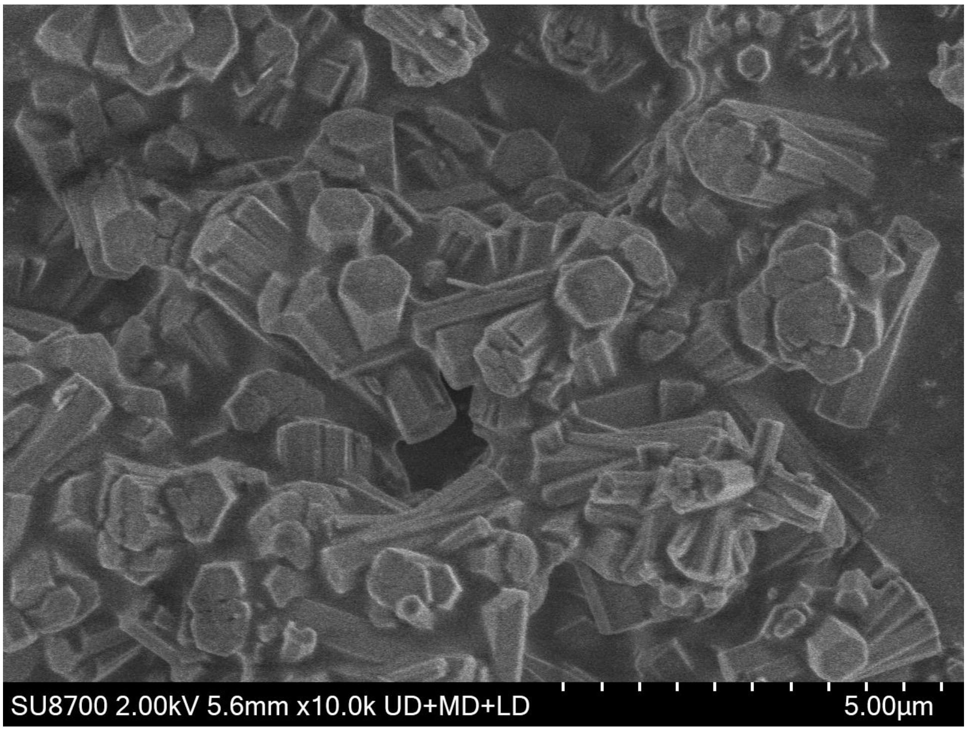

Figure S6: Scanning electron micrograph of **CAU-63-NO_3** at a magnification factor of 10K.

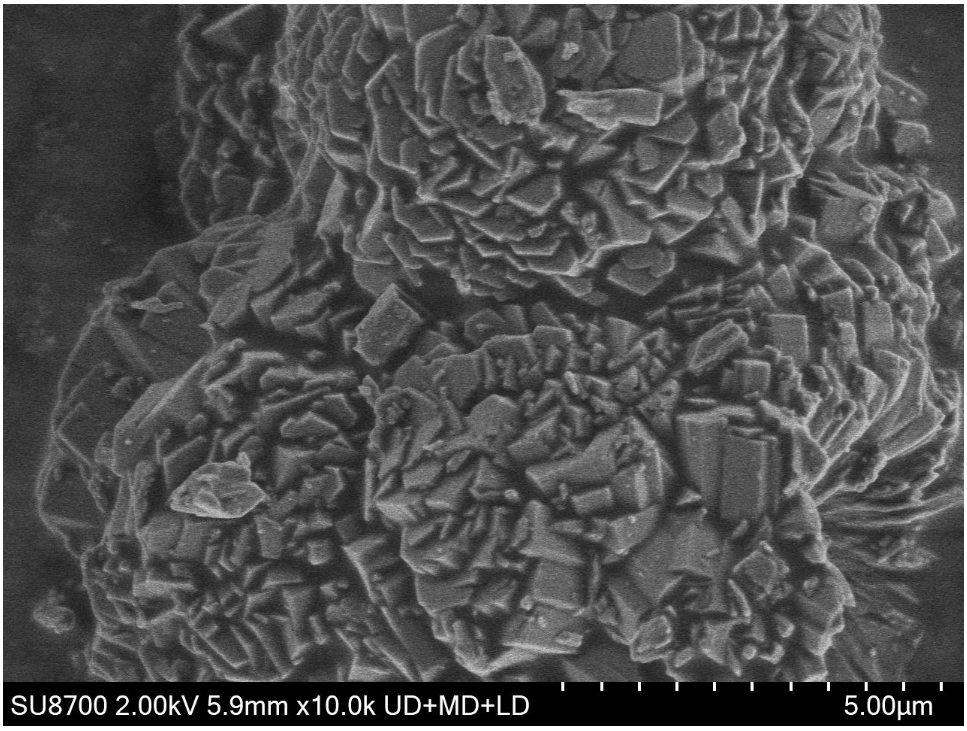

Figure S7: Scanning electron micrograph of **CAU-63-SO_4** at a magnification factor of 10K.

To assess the amount of chloride and sulfate ions contained in **CAU-63-Cl** and **CAU-63-SO_4** respectively, Energy-Dispersive-X-Ray (EDX) measurements were carried out to determine the molar ratio of aluminum to chloride/sulfur. For each sample at least 15 different crystallites were measured and the average ratio of Al/Cl and Al/S was determined to ensure a representative result. The amount of nitrate ions could not be determined via EDX since the instrument was not sensitive enough to measure nitrogen reliably. Therefore, the nitrate was quantified through elemental analysis.

Table S2: Results of the EDX measurements of **CAU-63-Cl** on different crystallites.

**CAU-63-Cl**	**Al/Cl**
**Site 1**	2.97
**Site 2**	2.20
**Site 3**	2.84
**Site 4**	3.02
**Site 5**	3.08
**Site 6**	3.03
**Site 7**	3.09
**Site 8**	3.07

**Site 9**	3.30
**Site 10**	2.81
**Site 11**	2.43
**Site 12**	2.76
**Site 13**	3.09
**Site 14**	3.08
**Site 15**	3.78
**Site 16**	3.09
**Average**	2.98 ±0.34

Table S3: Results of the EDX measurements of **CAU-63-SO_4** on different crystallites.

CAU-63-SO_4	Al/S
**Site 1**	6.22
**Site 2**	6.84
**Site 3**	7.00
**Site 4**	7.00
**Site 5**	7.37
**Site 6**	6.93
**Site 7**	7.46
**Site 8**	6.99
**Site 9**	5.74
**Site 10**	6.71
**Site 11**	6.57
**Site 12**	6.79
**Site 13**	6.70
**Site 14**	7.00
**Site 15**	6.37
**Site 16**	5.38
**Site 17**	6.71
**Site 18**	6.24
**Site 19**	5.92
**Average**	6.63±0.52

The Al/Cl ratio for **CAU-63-Cl** was determined to be 2.98 ±0.34 and the Al/S ratio for **CAU-63-SO4** to be 6.63±0.52. This leads to the compositions $[Al_7(OH)_{12}O_3(2,4\text{-}HPydc)_3] \cdot 2.35\pm0.26\ HCl$ and $[Al_7(OH)_{12}O_3(2,4\text{-}HPydc)_3] \cdot 1.06\pm0.08\ H_2SO_4$. For **CAU-63-NO3** and **CAU-63-SO4** the pore content was also determined through elemental analysis.

Table S4: Results of the elemental analysis of the **CAU-63-X** compounds.

	C [wt%]	H [wt%]	N [wt%]	S [wt%]
CAU-63-Cl	22.5	4.0	3.8	0
CAU-63-NO_3	24.81	3.26	6.03	0
CAU-63-SO_4	22.45	3.60	3.49	2.96

This corresponds to 1.38 NO_3 and 1.04 SO_4 per formula unit of **CAU-63** based on the ratio of N/C and S/C respectively. The amount of sulfate is therefore in very good agreement with the value derived from EDX measurements.

S4 Determination of the composition

This section contains the thermogravimetric (TG) and elemental analysis data, which were used to calculate the composition of the compounds and to compare these values to the ones deduced from the crystal structures.

CAU-63-Cl, $[Al_7(OH)_{12}O_3(2,4\text{-}HPydc)_3] \cdot 2.4\ HCl$

The TG curve of **CAU-63-Cl** shows two mass losses. The first corresponds to 12 % of the initial mass and can be correlated with the desorption of the water molecules from the pores The second mass loss of 59 % begins at around 250 °C and can be assigned to the decomposition of the framework.

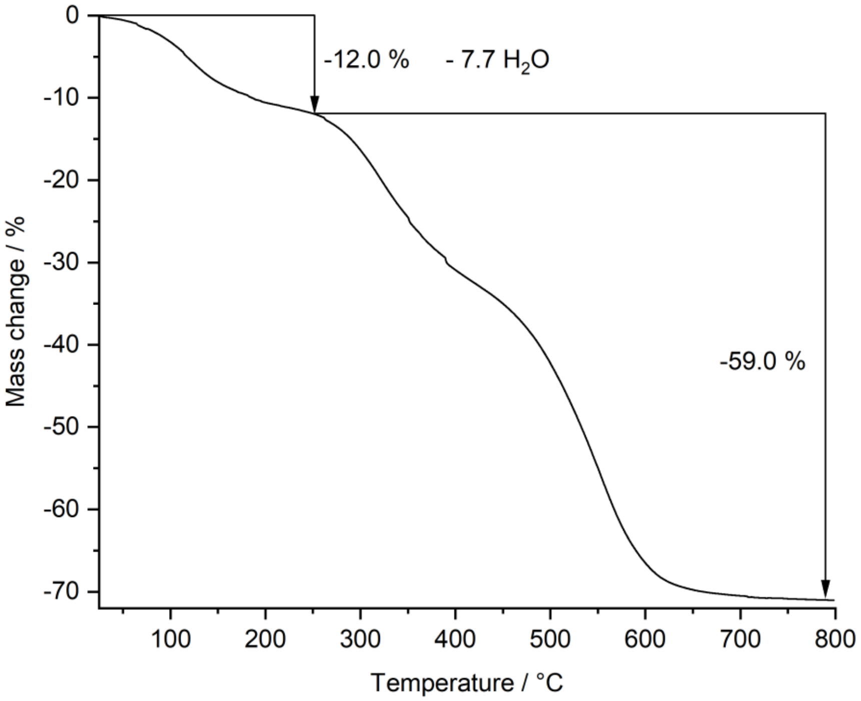

Figure S8: TG curve of **CAU-63-Cl**.

Table S5: Comparison of the calculated with the experimental composition of **CAU-63-Cl** using data from elemental and TG analysis.

	C / %	H / %	N / %	1. TG step / % 24-250 °C	2. TG step / % 250-800 °C
Experimental	22.5	4.0	3.8	12.0	59.0
$[Al_7(OH)_{12}O_3(2,4\text{-}HPydc)_3] \cdot 7.7\ H_2O \cdot 2.4\ HCl$	21.6	3.6	3.6	11.9	57.5

Al-Pydc-CP1 [$Al_2(OH)_5$(2,4-HPydc)]

The TG curve of **Al-Pydc-CP1** shows two distinct mass losses. The first starts at 260 °C and corresponds to 15.4 % of the initial mass. This correlates well with the loss of one CO_2 molecule, which could be the result of a decarboxylation reaction. The second mass loss of 50.1 % starts at 335 °C and corresponds to the decomposition of the structure.

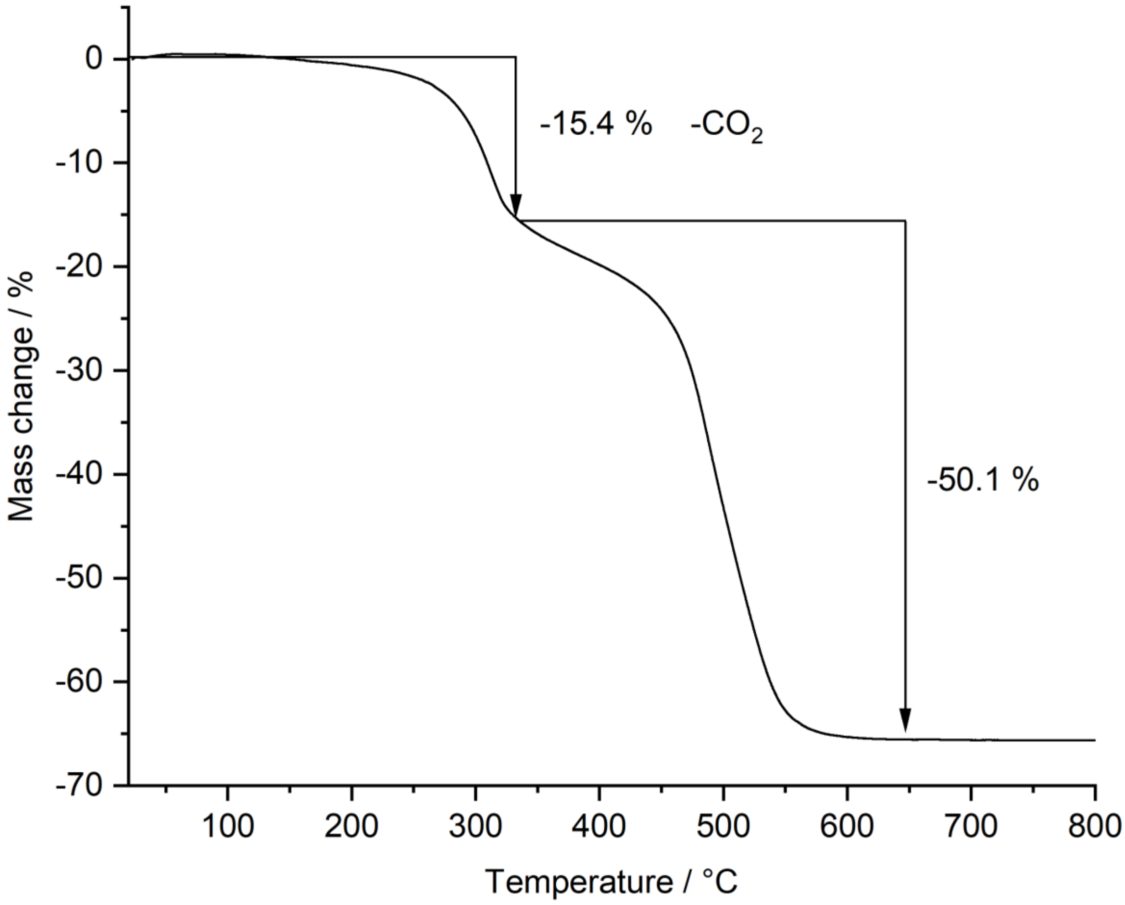

Figure S9: TG curve of **Al-Pydc-CP1**.

Table S6: Comparison of the calculated with the experimental composition of **Al-Pydc-CP1** using data from elemental and thermogravimetric analysis.

	C / %	H / %	N / %	1. TG step / % 24-335 °C	2. TG step / % 335-650 °C
Experimental	27.0	3.5	4.3	15.4	50.1
[$Al_2(OH)_5(C_7NH_4O_4)$]	27.6	3.0	4.6	14.4	52.2

Al-Pydc-CP2 [Al(OH)(H_2O)(2,4-Pydc)]

The TG curve of **Al-Pydc-CP2** shows two separate steps. The first mass loss of 9.8 % starts at around 240 °C and corresponds to the desorption of the coordinating water molecule from the structure (7.9 %). At around 350 °C, the framework decomposition sets in with a mass loss of 69.5 %.

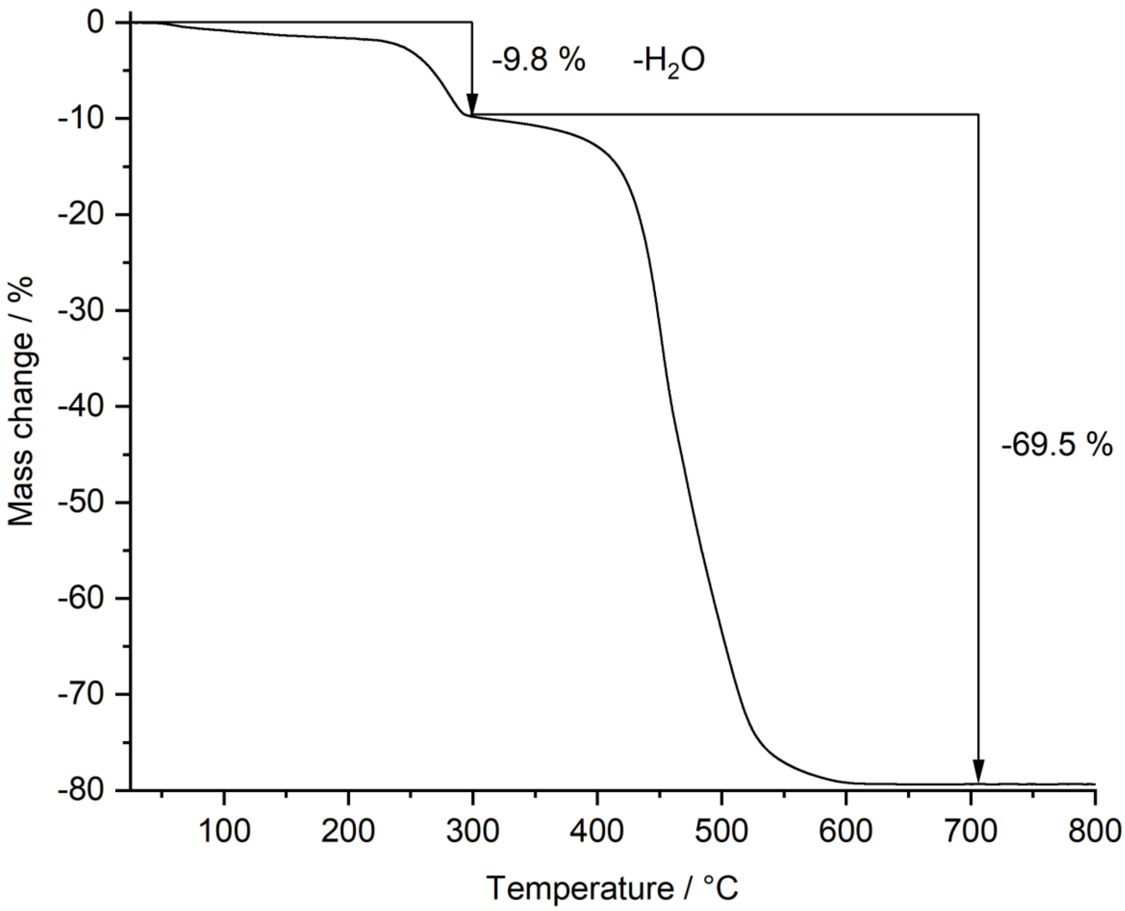

Figure S10: TG curve of **Al-Pydc-CP2**.

Table S7: Comparison of the calculated with the experimental composition of **Al-Pydc-CP2** using data from elemental and TG analysis.

	C / %	H/ %	N / %	1. TG step / % 24-300 °C	2. TG step / % 300-700 °C
Experimental	36.5	3.1	6.2	9.8	69.5
[Al(OH)(H_2O)($C_7NO_4H_3$)]	37.0	2.7	6.2	7.9	69.6

S5 IR spectroscopy

IR spectroscopy was carried out to further characterize the compounds, especially the presense of water molecules and the protonation state of the lutidinate ions. The assigned vibration bands are marked with an asterisk in the respective spectra.

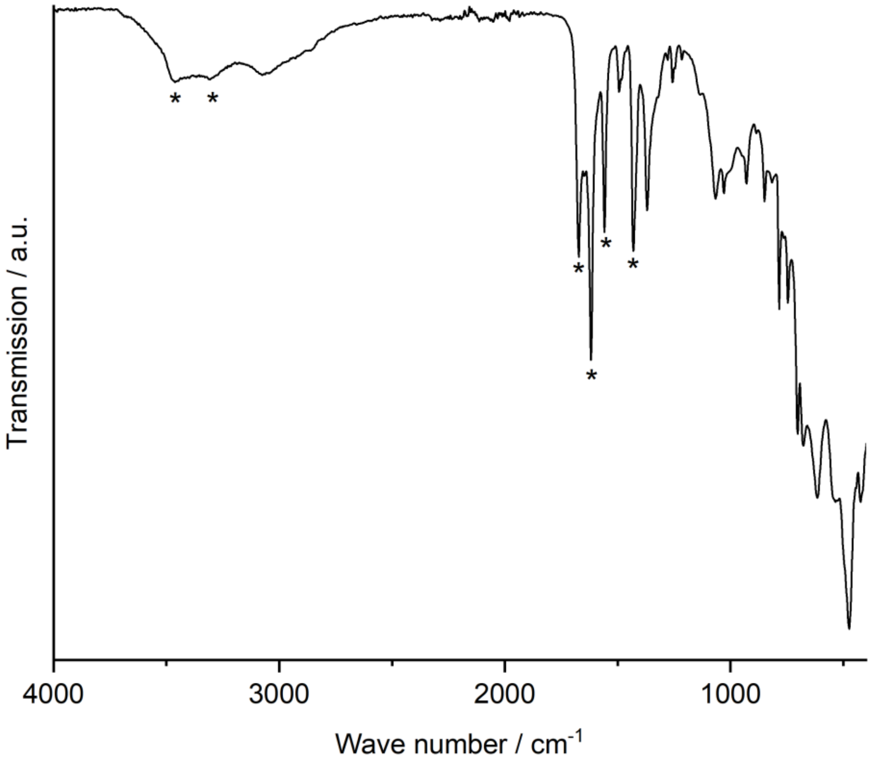

Figure S11: IR spectrum of **CAU-63-Cl**.

Table S8: Assignment of selected vibrations in the IR spectrum of **CAU-63-Cl**.[3,4]

**Wave number / cm^{-1}**	**Vibration**
**3464**	Hydrogen bonded O-H stretching
**3303**	Hydrogen bonded O-H stretching
**1672**	COOH C=O stretching
**1617**	COO^- asymmetric COO^- stretching
**1557**	Pyridine ring stretch
**1431**	Pyridine C=C and C=N in-plane

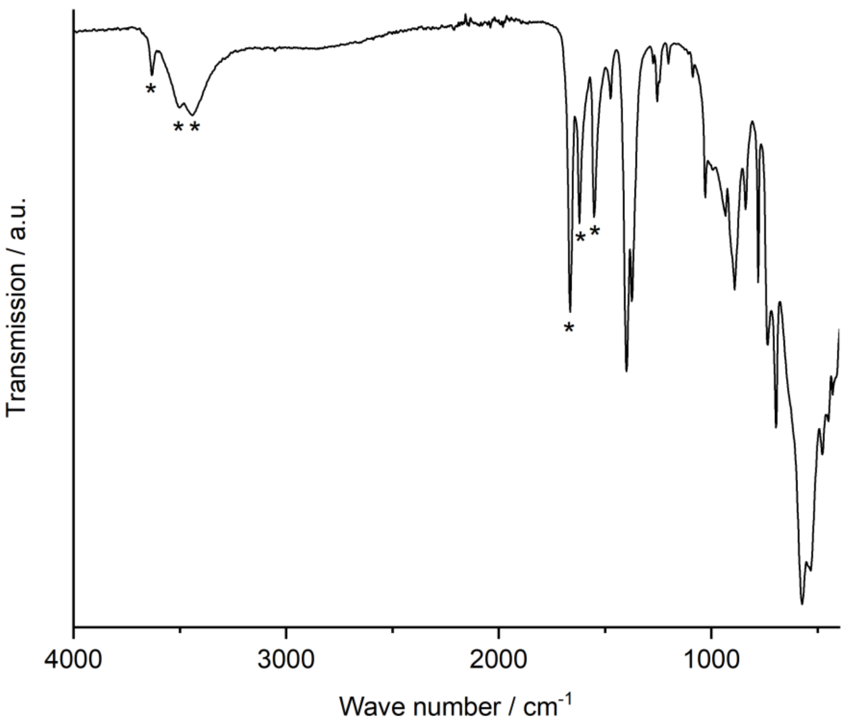

Figure S12: IR spectrum of **Al-Pydc-CP1**.

Table S9: Assignment of selected vibrations in the IR-spectrum of **Al-Pydc-CP1**.[3,4]

**Wave number / cm^{-1}**	**Vibration**
**3630**	O-H stretching
**3497**	Hydrogen bonded O-H stretching
**3430**	Hydrogen bonded O-H stretching
**1666**	COOH C=O stretching
**1621**	COO^- asymmetric COO^- stretching
**1549**	Pyridine ring stretch

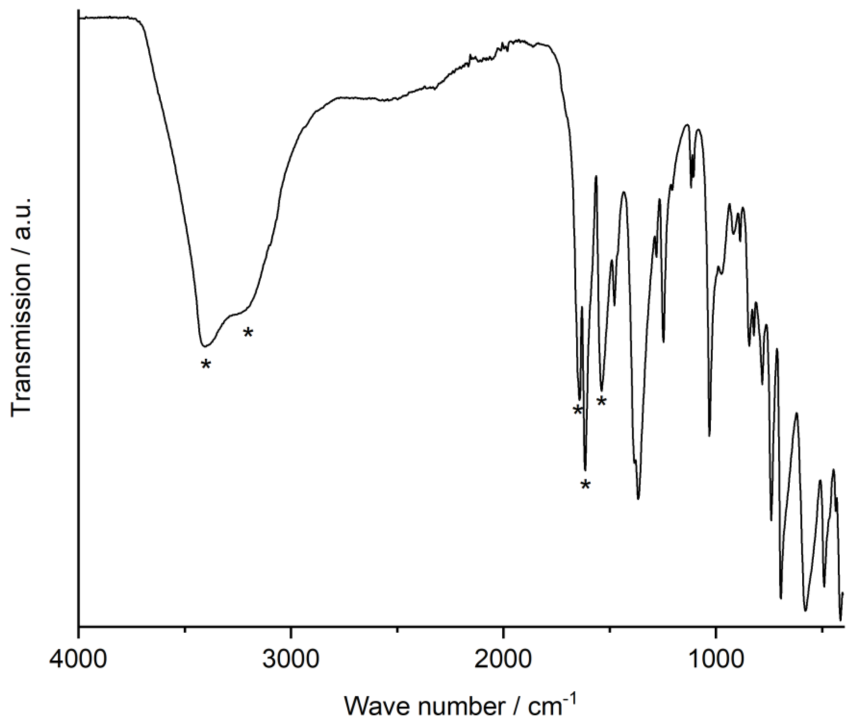

Figure S13: IR spectrum of **Al-Pydc-CP2**.

Table S10: Assignment of selected vibrations in the IR spectrum of **Al-Pydc-CP2**.[3,4]

**Wave number / cm^{-1}**	**Vibration**
**3403**	Hydrogen bonded O-H stretching
**3238**	Hydrogen bonded O-H stretching
**1641**	COO^- asymmetric COO^- stretching
**1615**	COO^- asymmetric COO^- stretching
**1537**	Pyridine ring stretch

S6 Structure determination and refinement

S6.1 Structure determination

Structure solution by 3D ED

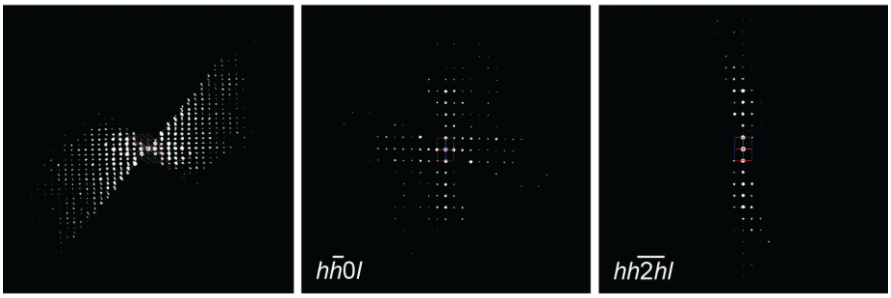

Figure S14: Reconstructed 3D reciprocal lattice (left) and two 2D slices *h*-*h*0*l* and *hh*-2*hl* of **CAU-63-Cl**.

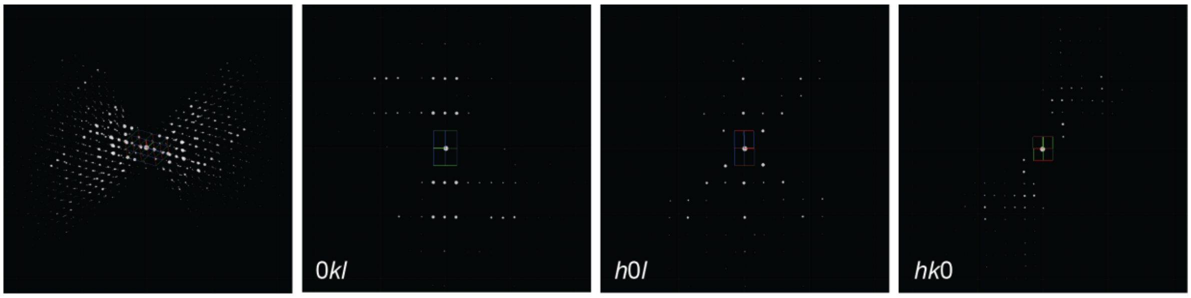

Figure S15 Reconstructed 3D reciprocal lattice (left) and three 2D slices 0*kl*, *h*0*l* and *hk*0, showing systematic absence: 0*kl* : *l=2n*, *h*0*l* : *h=2n*, *h*00 : *h=2n* and 00*l* : *l=2n* of **Al-Pydc-CP1**.

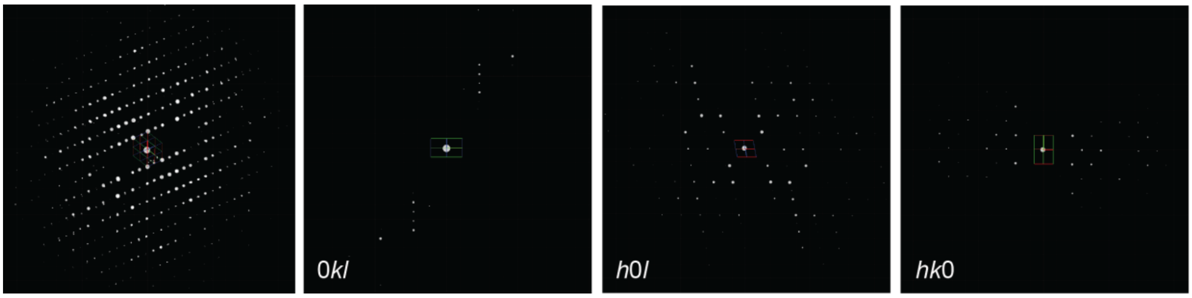

Figure S16: Reconstructed 3D reciprocal lattice and three 2D slices 0*kl*, *h*0*l* and *hk*0, showing systematic absence: 0*kl* : *k=2n*, *h*0*l* : *h=2n*, *l=2n*, *hk*0 : *h* + *k=2n* and 0*k*0 : *k=2n* of **Al-Pydc-CP2**.

Table S11: 3D ED data collection details.

	**CAU-63-Cl**	**Al-Pydc-CP1**	**Al-Pydc-CP2**
Wavelength, Å		0.2508	
Tilt range, °	89.17	107.81	99.37
Tilt step per frame, °		0.2300	
Exposure time/frame, s		0.5	
No. cRED frames	346	416	389
Resolution, Å	0.95	0.8	0.8
Observed reflections	4368	4039	3140
Unique reflections	1887	1949	1376
Completeness, %	98.2	88.1	70.5

3D ED data for **CAU-63-Cl**, **Al-Pydc-CP1** and **Al-Pydc-CP2** was collected at room temperature. Initially, the 3D reciprocal space was reconstructed in the software REDp, and the space group was determined based on reflection conditions from 2D cuts (see Figures S14-S16). Then, the 3D ED data was processed using the software XDS where the reflections were found, indexed and integrated. The data quality was evaluated based on the statistics, such as the I/σ value, CC(1/2), and completeness of data. The data counts as a signal if the signal-to-noise (or I/σ) ratio is bigger or equal to 1. The CC(1/2) statistic measures the correlation between intensities from random half-datasets, and when it is higher than 80% and there is a*, the data was included.

The structure solution for **CAU-63-Cl**, **Al-Pydc-CP1** and **Al-Pydc-CP2** was performed from single datasets using SHELXT. All non-hydrogen atoms of each structure were found *ab initio*. However, due to the similarity of scattering factors some atoms needed to be reassigned. Also, few hydrogen atoms can be resolved from the difference electrostatic potential Fourier map using SHELXL via OLEX2 software. The atoms were assumed assigned right if the shift value converged to 0.0.

S6.2 Rietveld refinements

The space group, the lattice parameter and the structural models of **CAU-63-Cl**, **Al-Pydc-CP1** and **Al-Pydc-CP2** were obtained from 3D-ED (Section S6.1). These models were refined against powder X-ray diffraction data to confirm the structure and phase purity of the bulk material. Firstly, Pawley refinements (Figure S17-S19) were performed on the PXRD patterns to refine background, cell parameters, diffractometer zero error and profile function (Simple_Axial_Model). The following R_{wp} values resulted from the Pawley refinements: 2.22 % (CAU-63-Cl), 4.59 % (**Al-Pydc-CP1**) and 1.50 % (**Al-Pydc-CP2**).

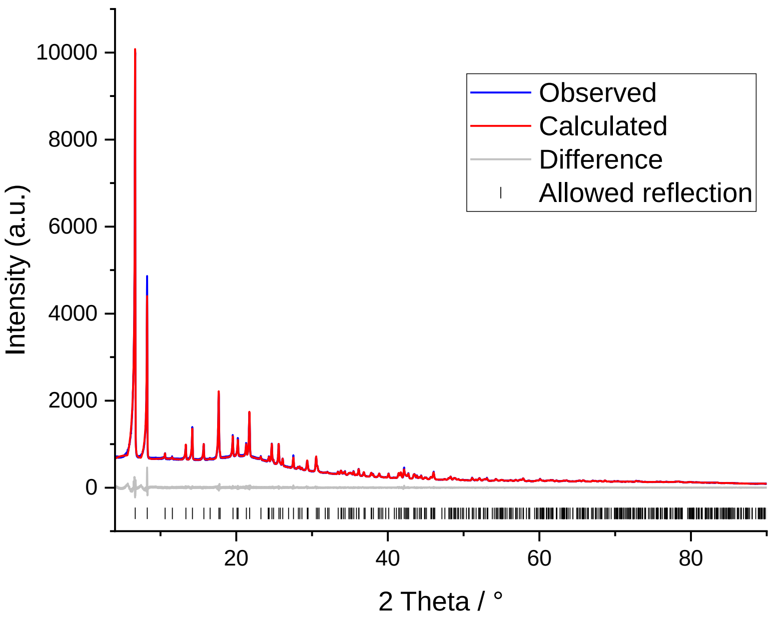

Figure S17: Final plot of the Pawley refinement of the PXRD pattern of **CAU-63-Cl** in the $P\overline{3}$ space group (R_{wp} = 2.22 %).

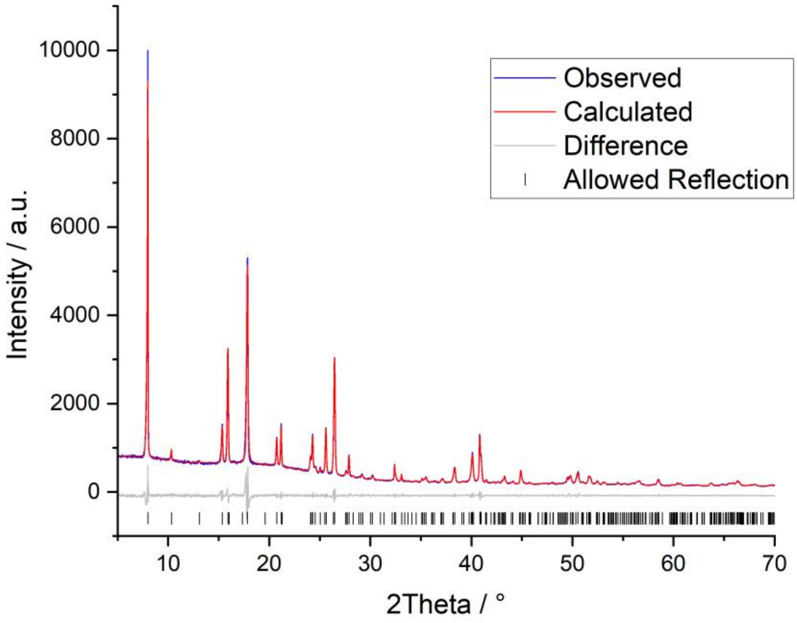

Figure S18: Pawley refinement of the PXRD pattern of **Al-Pydc-CP1** in the $Pca2_1$ space group (R_{wp} 4.59).

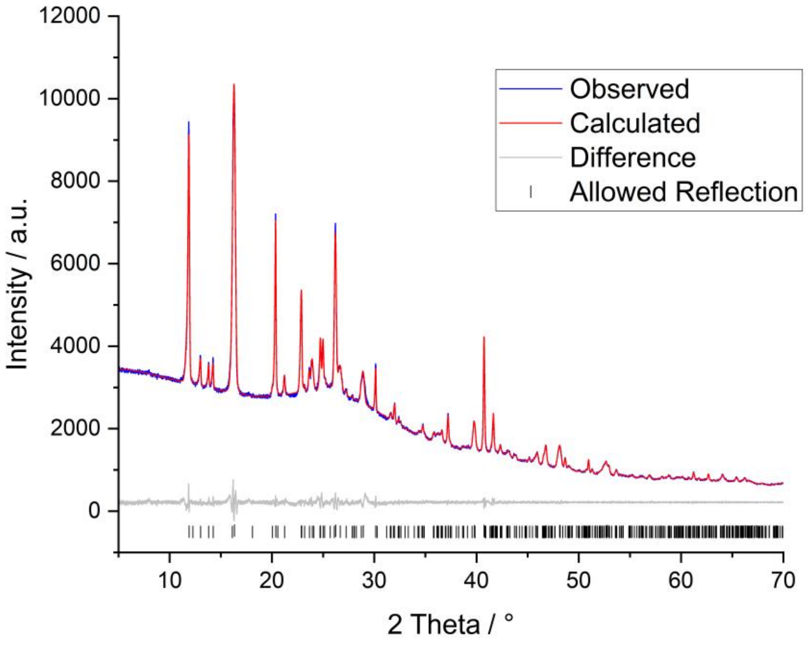

Figure S19: Pawley refinement of the PXRD pattern of **Al-Pydc-CP2** in the *C*2/*c* space group (R_{wp} 1.50).

In **CAU-63**, the asymmetric unit contains three independent aluminum atoms (Al00, Al01 and Al02), Al00 was found on the special position (⅓,⅔,z) while Al01 and Al02 are occupying general positions. The structural model was refined using distance and angle restrains, the Al-O bond length was set to 1.95 Å and the Al-N distance to 2.07 Å. Given the complexity of the IBU a part of the dimer formed by the atoms Al01 and Al02 was modelled as a rigid body as shown in Figure S20.

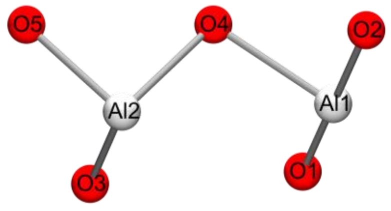

Figure S20: Representation of the rigid body used to model a part of the IBU. The aluminum atoms are depicted in grey and the oxygen atoms in red.

A certain degree of freedom (10°) was given to the angles in the rigid body. The asymmetric unit also contains one linker that was modeled as a Z matrix. The statistical weight of these restraints was maintained high in the first stages of the refinement and then progressively decreased and the position were left free to refine. In addition, the thermal factors (b_{eq}) were refined in three independent groups, one for the light atoms (C, O, N), one for the Al atoms and one for the guest atoms (Cl). Since the electron density attributed to the Cl atoms inside the

pores of CAU-63-Cl is delocalized, we refined this by using three independent Cl atoms with fractional occupancies limited to 0.3 each, based on the Cl content obtained from EDX measurements. At the end of the refinement, all the parameters were refined together until convergence and the shifts in all parameters were lower than their standard deviations. The Rietveld plot and a selection of bond distances and bond angles are reported in Figure S21, Table S12 and Table S13 respectively.

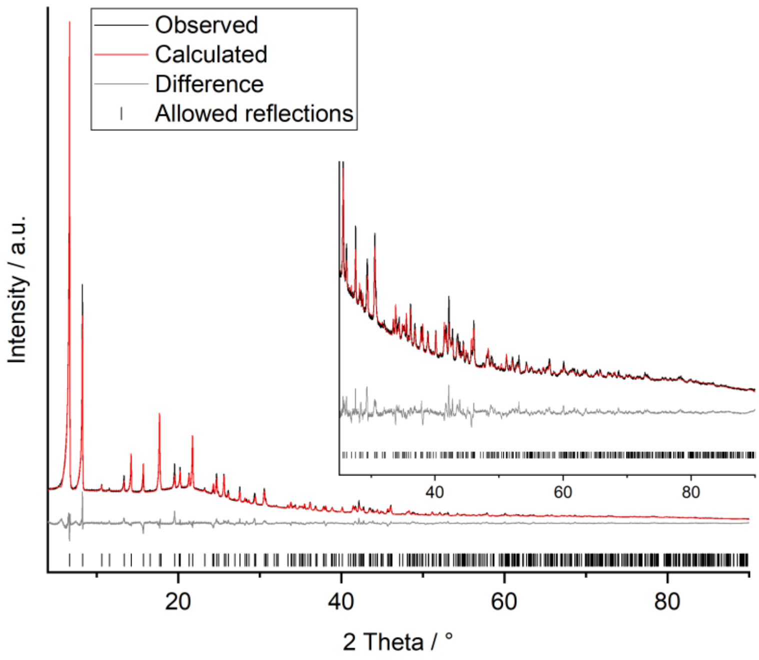

Figure S21: Results of the Rietveld refinement of **CAU-63-Cl**. Calculated PXRD pattern (red) and the observed pattern in black. The grey line shows the difference between the two graphs and the black ticks show the possible reflection positions.

Table S12: Selected bond distances for **CAU-63-Cl**.

Bond	Distance (Å)	Bond	Distance (Å)
Al00-O14	1.756(4)	C1-C6	1.410(6)
Al00-O2	1.919(13)	C1-C8	1.550(10)
Al1-O2	1.878(19)	C2-C3	1.410(6)
Al1-O1	1.96(3)	C2-C1	1.410(6)
Al1-O4	1.969(3)	C3-N1	1.376(3)
Al1-O13	2.002(7)	C3-C7	1.550(10)
Al1-O3	2.043(4)	C5-N1	1.376(3)
Al2-O11	1.724(6)	C5-C6	1.410(6)
Al2-O2	1.83(3)	C7-O12	1.266(4)
Al2-O4	1.956(5)	C7-O11	1.266(4)
Al2-O5	1.97(2)	C8-O13	1.266(6)
Al2-O1	2.00(2)	C8-O14	1.266(6)
Al2-N1	2.377(2)		

Table S13: Selected bond angles for **CAU-63-Cl**.

Bond angle	Angle (°)	Bond angle	Angle (°)
O14-Al00-O14	86.88(16)	O1-Al1-O3	90.1(6)
O2-Al00-O14	95.04(18)	O1-Al1-O13	178.1(8)
O2-Al00-O14	90.6(4)	O3-Al2-O11	175.08(16)
O2-Al00-O14	176.7(3)	O4-Al2-O3	89.9444(3)
O2-Al00-O2	87.6(5)	O4-Al2-O11	88.0(3)
O2-Al00-O14	90.6(4)	O5-Al2-O4	86.62(2)
O13-Al00-O14	123.02(16)	O5-Al2-O3	89.06(5)
O1-Al1-O2	175.1(6)	O5-Al2-O11	86.35(15)
O4-Al1-O1	89.9(6)	O1-Al2-O5	173.8(4)
O4-Al1-O2	95.00(15)	O1-Al2-O4	88.41(8)
O13-Al1-O4	92.2(3)	O1-Al2-O3	87.2(7)
O13-Al1-O1	95.3(2)	O1-Al2-O11	97.2(8)
O13-Al1-O2	84.5(2)	N1-Al2-O1	104.15(10)
O3-Al1-O13	91.2(2)	N1-Al2-O5	80.63(4)
O3-Al1-O4	172.2(7)	N1-Al2-O4	167.03(5)
O3-Al1-O1	82.8(9)	N1-Al2-O3	87.53(7)
O3-Al1-O2	92.3(7)	N1-Al2-O11	93.5(3)

For **Al-Pydc-CP1**, the structural model was refined using distance and angle restrains. The following restrains on the bond lengths were used: Al-O = 1.95 Å, Al-N = 2.07 Å. The penalties of these restraints were maintained high at the first stages of the refinement and then progressively decreased. The asymmetric unit contains two independent Al atoms (Al0 and Al1), four μ_2-O and one terminal oxygen atom in general positions and one linker molecule modelled as a rigid body using a Z-matrix. The coordinates (*x,y,z*) of the atoms in general position as well as the coordinates Z-matrix were let free to refine. The thermal factors (b_{eq}) were refined in two independent groups, one for the light atoms (C, O, N) and one for the Al atoms, then left free to refine. At the end of the refinement, all the parameters were refined together until convergence and the shifts in all parameters were lower than their standard deviations. The Rietveld plot and a selection of bond distances are reported in Figure S22 and Table S14 respectively.

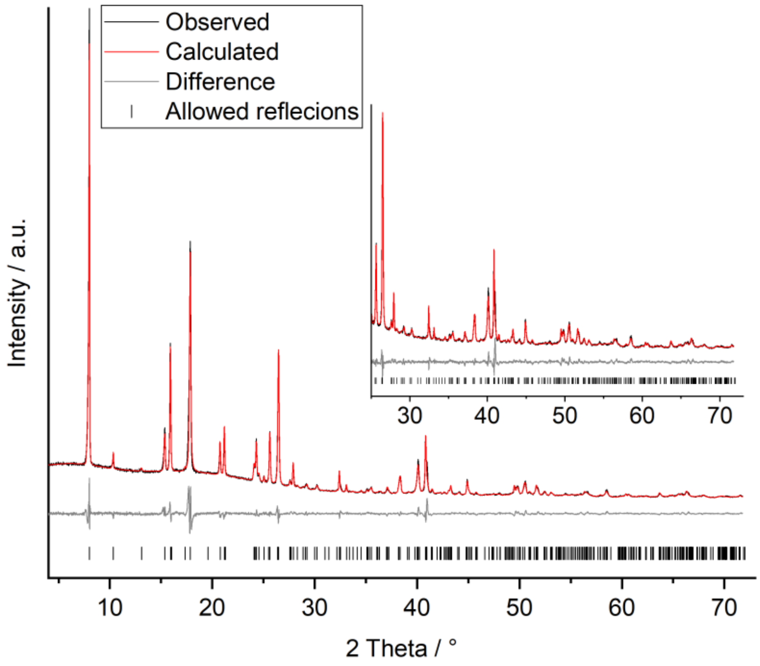

Figure S22: Comparison of the calculated XRD-pattern of **Al-Pydc-CP1** from Rietveld refinement based on a structure model from 3D-electron diffraction (red) with the observed pattern in black. The grey line shows the difference between the two graphs and the black ticks show the possible reflection positions.

Table S14: Selected bond distances for **Al-Pydc-CP1**.

Bond	Distance (Å)	Bond	Distance (Å)
Al1-Om2	1.82(2)	C1-C2	1.370(11)
Al1-O3	1.86(4)	C1-C6	1.52(2)
Al1-Om4	1.92(3)	C2-C1	1.370(11)
Al1-Om1	1.93(3)	C2-C3	1.370(11)
Al1-Om3	1.94(3)	C3-C4	1.370(11)
Al1-OH	1.96(3)	C3-C7	1.52(2)
Al2-Om2	1.91(3)	C4-C5	1.370(11)
Al2-Om1	1.93(3)	C5-N1	1.355(13)
Al2-Om3	1.927(19)	C6-O1	1.30(6)
Al2-O1	1.98(10)	C6-O2	1.30(6)
Al2-Om4	2.05(3)	C7-O4	1.30(7)
C1-N1	1.355(13)	C7-O3	1.30(7)
Al2-N1	2.16(3)		

For **Al-Pydc-CP2**, the structural model was refined by using distance and angle restraints. The following restraints on the bond lengths were used: Al-O = 1.95 Å, Al-N = 2.07 Å. The penalties of these restraints were maintained high at the first stages of the refinement and then progressively decreased. The asymmetric unit contains two independent Al atoms (Al0 and Al1), located at the special positions (½, y_1, ¼) and (½, y_2, ¼), one bridging oxygen atom in a

general position and one linker molecule modelled as a rigid body using a Z-matrix. The coordinates (*x*,*y*,*z*) of the atoms in general position as well as the coordinates Z-matrix were left free to refine. The thermal factors (b_{eq}) were refined in two independent groups, one for the light atoms (C, O, N) and one for the Al atoms, then left free to refine. Stephens peak shape broadening macro[1] for monoclinic cell was applied to better model the peak shape. At the end of the refinement, all the parameters were refined together until convergence until the shifts in all parameters were lower than their standard deviations. The Rietveld plot and a selection of bond distances are reported in Figure S23 and Table S15 respectively.

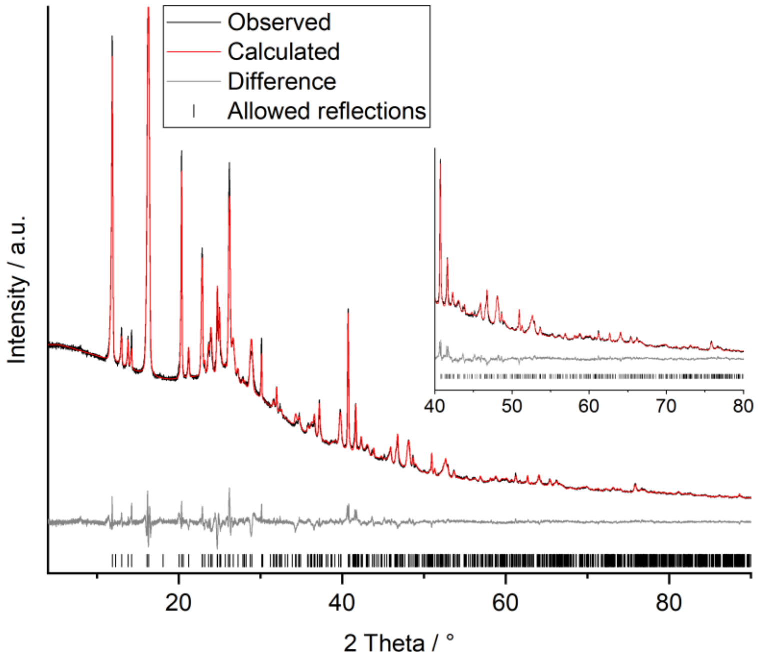

Figure 23: Comparison of the calculated XRD-pattern of **Al-Pydc-CP2** from Rietveld refinement based on a structure model from 3D-electron diffraction (red) with the observed pattern in black. The grey line shows the difference between the two graphs and the black ticks show the possible reflection positions.

Table S15: Selected bond distances for **Al-Pydc-CP2**.

Bond	Distance (Å)	Bond	Distance (Å)
Al0-O04	1.809(12)	C2-C1	1.370(3)
Al0-O2	2.04(3)	C2-C3	1.370(3)
Al0-N1	2.100(8)	C3-N1	1.370(3)
Al1-O07	1.895(11)	C3-C7	1.476(12)
Al1-O3	1.929(16)	N1-C5	1.370(3)
Al1-O04	2.022(12)	C5-C6	1.370(3)
Al1-O4	3.19(3)	C7-O1	1.30(3)
C1-C2	1.370(3)	C7-O2	1.30(3)
C1-C6	1.370(3)	C8-O3	1.30(2)
C1-C8	1.476(12)	C8-O4	1.30(2)

Table S16: Comparison of the lattice parameters obtained from ED and Rietveld refinement of **CAU-63-Cl**, **Al-Pydc-CP1** and **Al-Pydc-CP2**.

	**CAU-63-Cl**		**Al-Pydc-CP1**		**Al-Pydc-CP2**	
	**ED**	**Rietveld**	**ED**	**Rietveld**	**ED**	**Rietveld**
a (Å)	15.680(2)	15.2893(4)	13.540(3)	13.5097(6)	13.700(3)	13.0695(5)
b (Å)	15.680(2)	15.2893(4)	11.240(2)	11.04055(18)	9.1700(18)	8.7099(3)
c (Å)	11.130(2)	10.7076(3)	7.5000(15)	7.31878(14)	15.750(3)	15.1655(9)
α (°)	90	90	90	90	90	90
β (°)	90	90	90	90	101.41(3)	101.676(3)
γ (°)	120	120	90	90	90	90
V ($Å^3$)	2369.8(8)	2167.70(12)	1141.4(4)	1091.63(6)	1939.5(7)	1690.62(14)

S7 Details of the crystal structures

The following section contains some extra details about the crystal structures of the three compounds, including the coordination modes of the lutidinate ions. The positions of the hydrogen atoms could not be determined from PXRD data and the hydrogen atoms are therefore not shown.

S7.1 CAU-63-Cl: $[Al_7O_3(OH)_{12}(2,4\text{-}HPydc)_3] \cdot 2.4\ HCl$

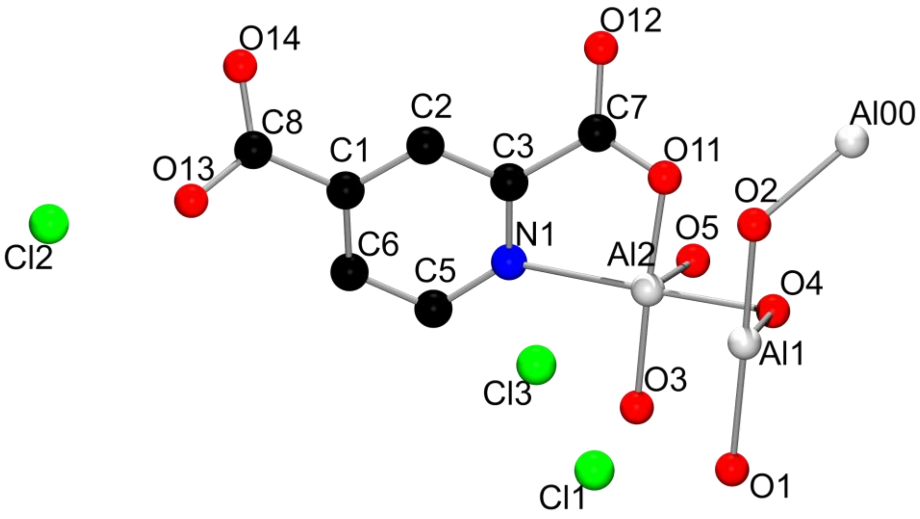

Figure S24: Asymetric unit of **CAU-63-Cl** including the chlorine atoms that are located in the pores of the structure.

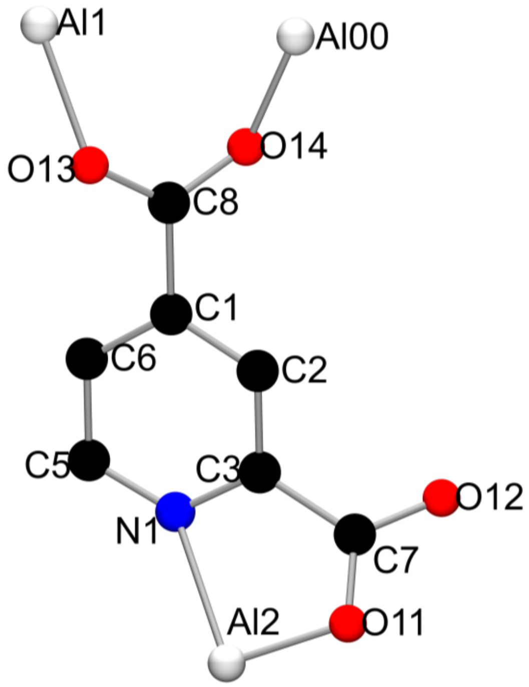

Figure S25: Coordination mode of the lutidinate ion in **CAU-63-Cl**.

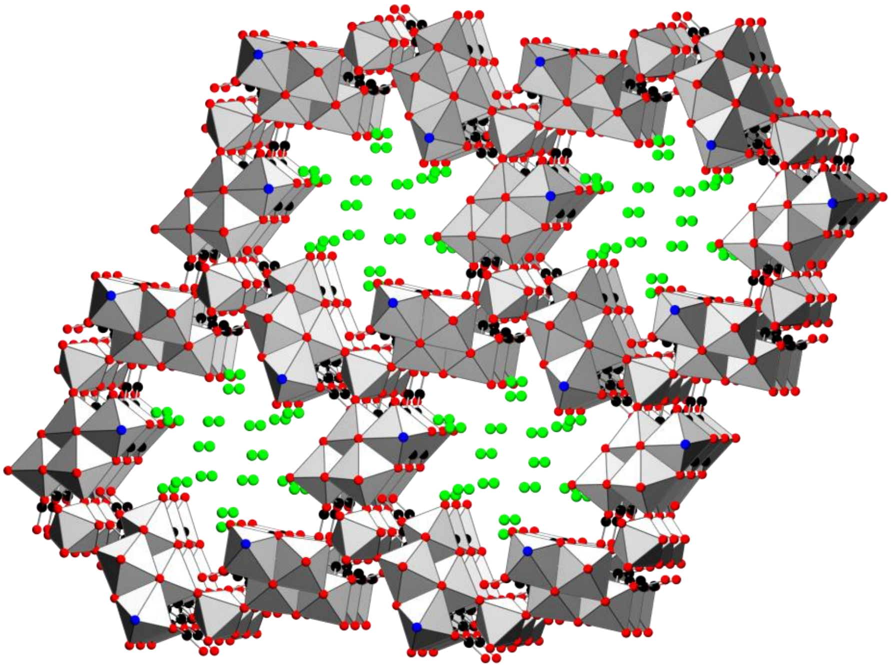

Figure S26: View of the channels of **CAU-63-Cl** with the trapped chloride ions along [001].

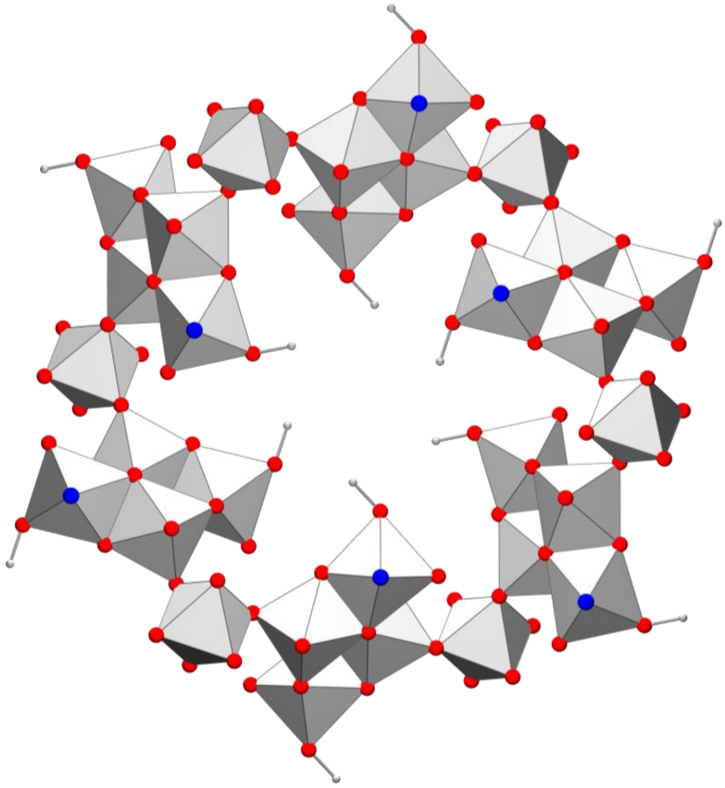

Figure S27: Pore window of **CAU-63-Cl** with the terminal OH-groups limiting the pore diameter based on the initial structure model from 3D-ED. The pore limiting diameter was calculated to be 3.07 Å using the Pore Analyzer module in Mercury 4.0[5] with a helium probe at a simulated temperature of 298 K. This value is in line with the pore window range of 2.9 Å to 3.3 Å obtained from sorption experiments.

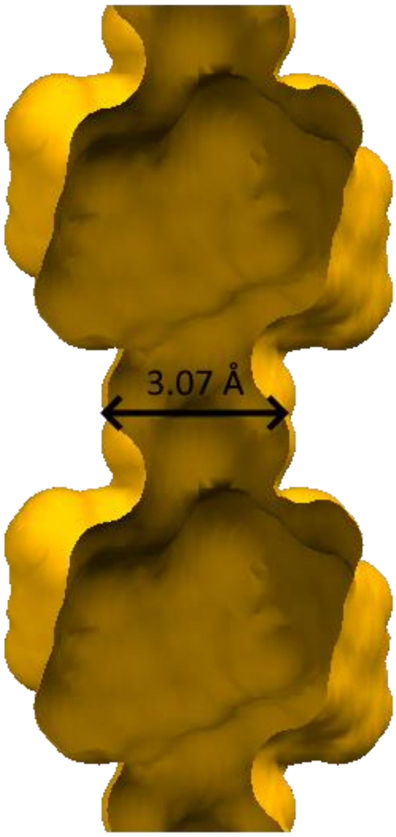

Figure S28: Cross section of the pore space of **CAU-63-Cl** with the pore limiting diameter of 3.07 Å calculated using Mercury 4.0[2] with a probe diameter of 1.2 Å.

S7.2 Al-Pydc-CP1: [$Al_2(OH)_5$(2,4-HPydc]

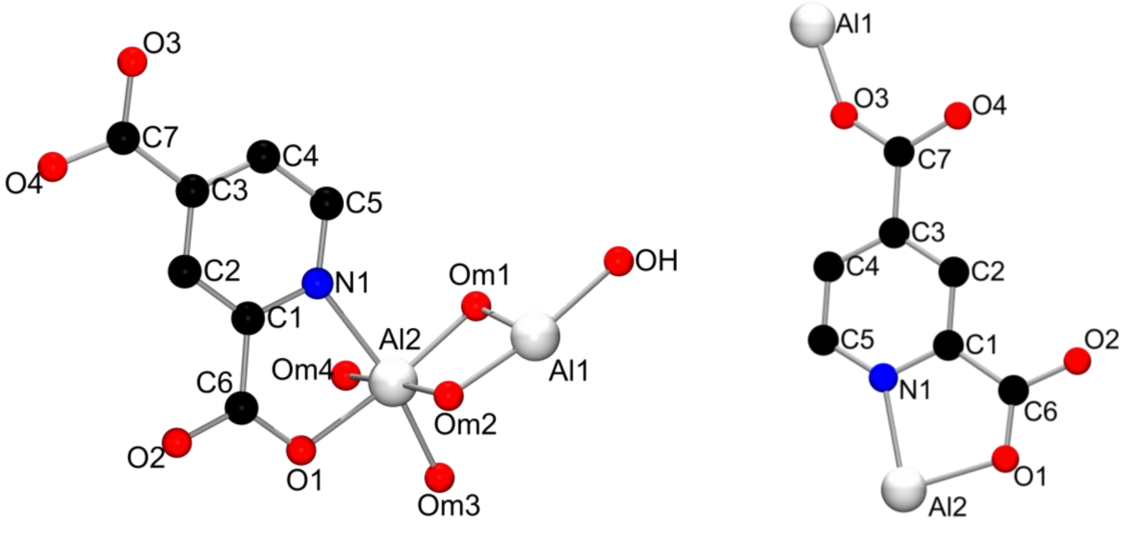

Figure S29: Asymmetric unit of **Al-Pydc-CP1** with atom labels and coordination of the lutidinate ion in **Al-Pydc-CP1**.

S7.3 Al-Pydc-CP2: [Al(OH)(H_2O)(2,4-Pydc)]

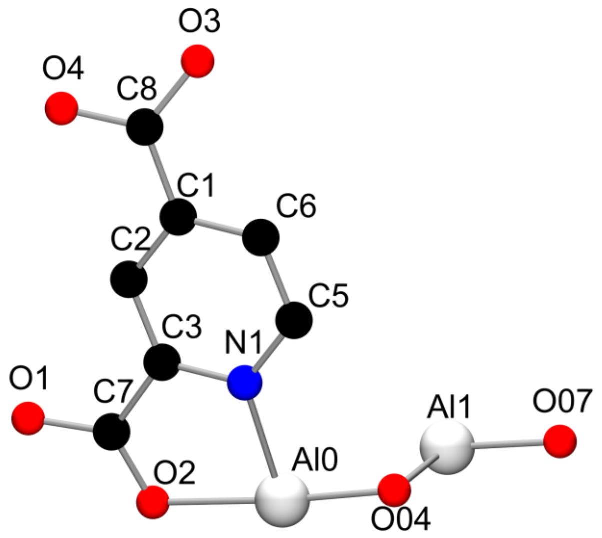

Figure S30: Asymmetric unit of **Al-Pydc-CP2** with atom labels.

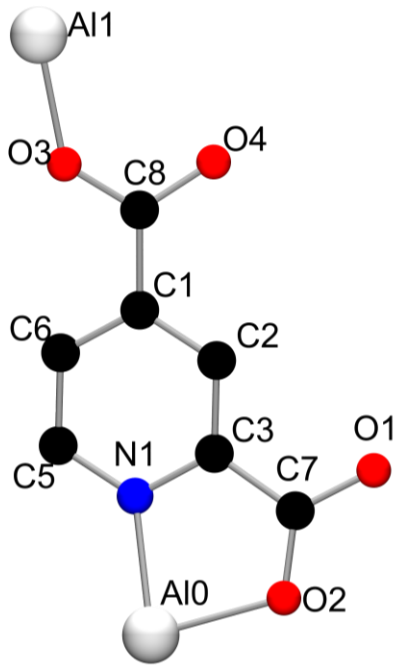

Figure S31: Coordination of the lutidinate ion in **Al-Pydc-CP2**.

S8 Sorption

The volumetric gas adsorption performance of **CAU-63-Cl** was measured at 77 K for N_2 and at 303 K for CO_2. In both cases, **CAU-63-Cl** shows negligible uptake and can therefore be considered non-porous towards either gas.

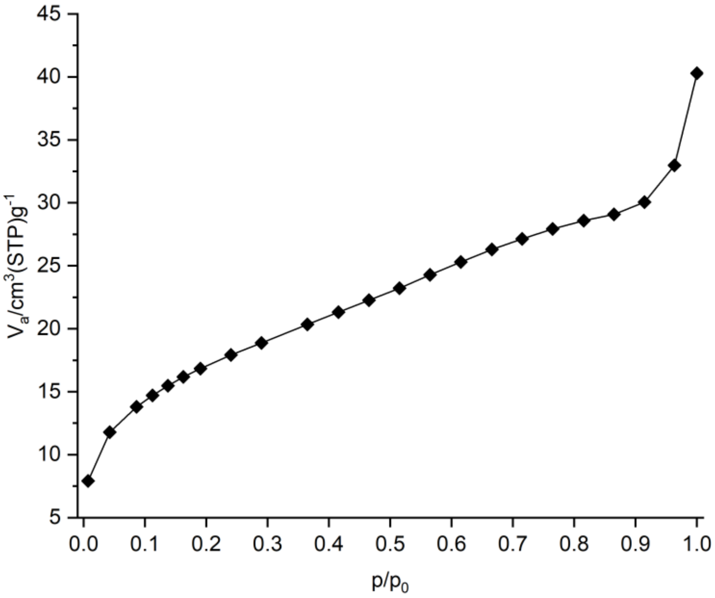

Figure S32: Nitrogen sorption isotherm of **CAU-63-Cl** at 77 K.

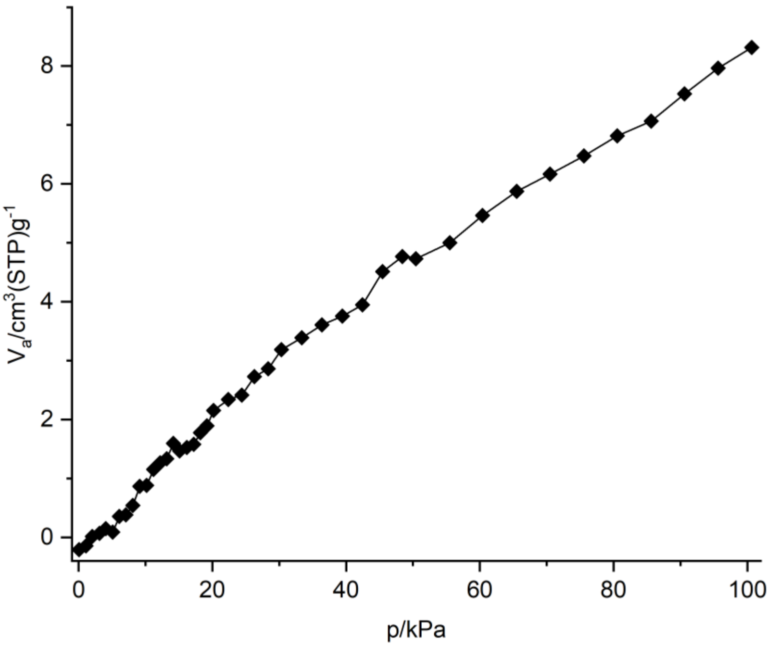

Figure S33: CO_2 adsorption isotherm of **CAU-63-Cl** at 303 K.

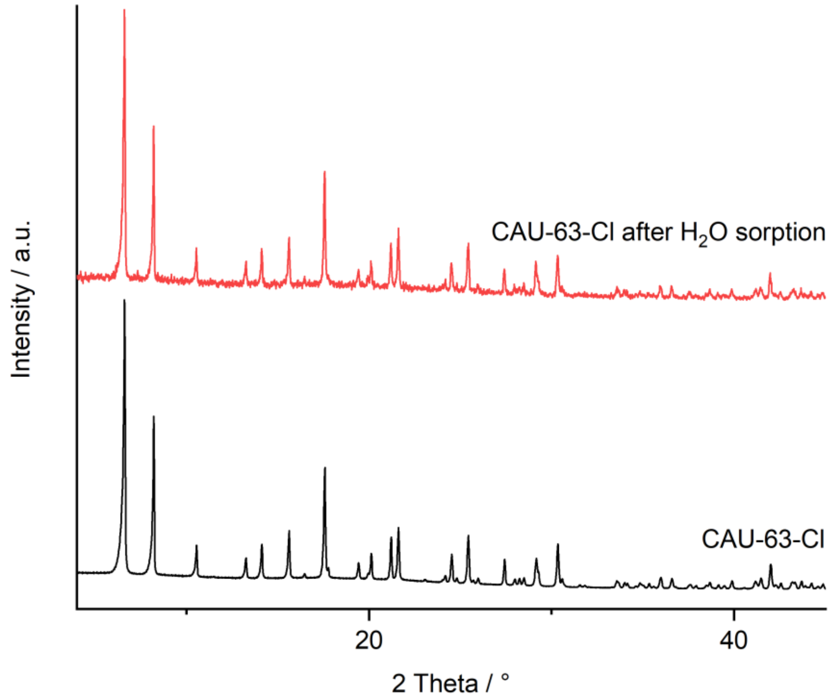

Figure S34: Comparison of the PXRD patterns of **CAU-63-Cl** before and after the H_2O sorption measurement at 298 K.

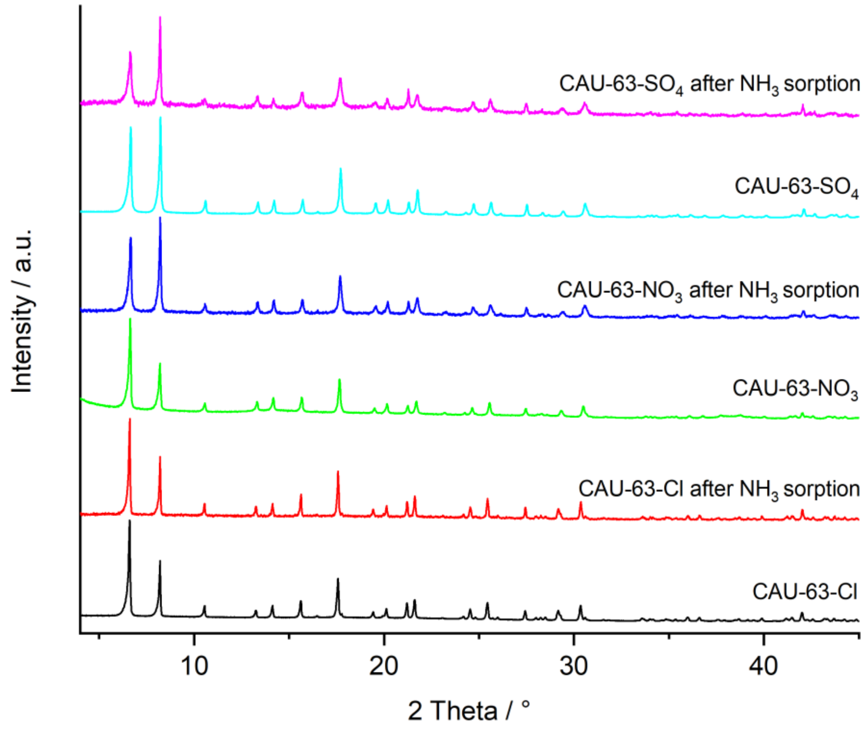

Figure S35: Comparison of the XRD-patterns of **CAU-63-Cl**, **CAU-NO_3** and **CAU-63-SO_4** before and after the NH_3 sorption. In all three cases the material retains its crystallinity but in the case of **CAU-NO_3** and **CAU-63-SO_4** the relative intensities of the first two reflections change, which could be explained by the different pore content after NH_3 exposure.

S9 References

(1) Stephens, P. W. Phenomenological model of anisotropic peak broadening in powder diffraction. *J. Appl. Crystallogr.* **1999**, *32*, 281–289.

(2) Macrae, C. F.; Sovago, I.; Cottrell, S. J.; Galek, P. T. A.; McCabe, P.; Pidcock, E.; Platings, M.; Shields, G. P.; Stevens, J. S.; Towler, M. *et al.* Mercury 4.0: From visualization to analysis, design and prediction. *J. Appl. Crystallogr.* **2020**, *53*, 226–235.

(3) Socrates, G. *Infrared and Raman characteristic group frequencies: Tables and charts*, 3rd ed.; John Wiley & Sons LTD: Chichester [etc.], 2015.

(4) Hesse, M.; Meier, H.; Zeeh, B. *Spektroskopische Methoden in der organischen Chemie*, 4. überarb. Aufl.; G. Thieme: Stuttgart, New York, 1991.
